# DeTOKI identifies and characterizes the dynamics of chromatin TAD-like domains in a single cell

**DOI:** 10.1186/s13059-021-02435-7

**Published:** 2021-07-27

**Authors:** Xiao Li, Guangjie Zeng, Angsheng Li, Zhihua Zhang

**Affiliations:** 1 CAS Key Laboratory of Genome Sciences and Information, Beijing Institute of Genomics, Chinese Academy of Sciences, and China National Center for Bioinformation, Beijing, 100101 China; 2 School of Life Science, University of Chinese Academy of Sciences, Beijing, People’s Republic of China; 3 School of Artificial Intelligence, University of Chinese Academy of Sciences, Beijing, People’s Republic of China; 4 State Key Laboratory of Software Development Environment, School of Computer Science, Beihang University, 100083 Beijing, People’s Republic of China

**Keywords:** 3D genome, Hi-C, TAD, Single cell

## Abstract

Topologically associating domains (TAD) are a key structure of the 3D mammalian genomes. However, the prevalence and dynamics of TAD-like domains in single cells remain elusive. Here we develop a new algorithm, named deTOKI, to decode TAD-like domains with single-cell Hi-C data. By non-negative matrix factorization, deTOKI seeks regions that insulate the genome into blocks with minimal chance of clustering. deTOKI outperforms competing tools and reliably identifies TAD-like domains in single cells. Finally, we find that TAD-like domains are not only prevalent, but also subject to tight regulation in single cells.

**Supplementary Information:**

The online version contains supplementary material available at 10.1186/s13059-021-02435-7.

## Background

The eukaryote genome in the nucleus is folded into a hierarchical configuration [1, 2], as revealed by imaging technologies [3] and chromosome conformation capture (3C)-based technologies [4–12], e.g., Hi-C [8]. The hierarchical configuration consists of chromosomal territories [8, 13, 14], A and B compartments [8], domain structures, such as topologically associating domains (TADs) [14, 15], compartment domains [16], or CTCF loop domains [17], and chromatin loops [17–19]. Such configurations have been routinely discussed in many studies [2]. TADs might be the most investigated chromatin feature in the literature since their disruption can cause severe diseases [20], including cancer [21].

TAD structures have been primarily revealed by Hi-C in bulk cells [8], while the existence and biogenesis of TADs in individual cells remain unclear. Super-resolution imaging data have shown the existence of and variations in the TAD-like domain structures in single cells [22]. Given the large cell-to-cell variations of chromatin architecture observed in individual cells, TADs could be a partially emergent property of a cell population. That is, the dynamics of chromatin in single cells per se may generate, at least in part, the TADs we observed in the bulk cells [23–25]. Since the origin and dynamics of TADs are keys to understanding gene regulation [26, 27], unraveling the nature of single-cell TAD-like domain structure is essential. However, the systematic survey of TAD-like domain structure, including its dynamics, in single cells remains a major challenge in the field.

A long list of TAD detection tools is available in the literature, and the methods are sophisticated and diverse [28, 29]. TADs were first identified using certain local genomic or topological features, e.g., the directionality index (DI) [14], the insulation score (IS) [30], the arrowhead score [18], IC-Finder [31], and ClusterTAD [32]. Later, methods based on probabilistic models with certain assumptions about the data distributions were developed, such as GMAP [33], PSYCHIC [34], HiCseg [35], TADbit [36], and TADtree [37]. Some other tools utilize dynamic programming to optimize a global object function, e.g., Armatus [38] and Matryoshka [39]. When treating the Hi-C matrix as a network connection matrix, an entire toolbox is available from graph theory, e.g., MrTADFinder, 3DnetMod [40, 41], and we recently developed deDoc [42]. However, comparisons have shown that almost none of them worked reliably with ultra-low-resolution Hi-C data [28, 43]. Among all TAD predictors, IS and deDoc worked the best with low-resolution Hi-C [30, 42]; however, TADs are virtually undetectable in experimental single-cell Hi-C data.

The inadequate handling of single-cell Hi-C data by current TAD prediction methods stems from the ultra-sparsity of chromatin interactions. A single cell has two copies for any given locus, which means that only two copies of Hi-C ligations, at most, could possibly exist in the single-cell Hi-C (scHi-C) libraries for that locus. Thus, the fluctuations from stochastic chromatin interactions per se, or from PCR proliferation, have exponential effect on the final scHi-C sequencing data. Consequently, to systematically survey TAD-like domain structure, we need a computational tool able to reliably process such ultra-sparse data from scHi-C.

Non-negative matrix factorization (NMF) consists of a group of algorithms in multivariate analysis whereby a non-negative matrix is factorized into two or more non-negative matrices [44]. The NMF has been widely used in processing single-cell omics data, e.g., coupled NMF [45]. The advantage of NMF is its low rank representation, which retrieves key information embedded in the noisy sparse data. As a sparse non-negative matrix, the sparsity issue of scHi-C data can also be solved by NMF. Therefore, we developed a new method using NMF to decode TAD boundaries that keep chromatin interaction isolated (deTOKI) from ultra-sparse Hi-C data. We present evidence that deTOKI can reliably predict TAD-like domain structures at the single-cell level. Further, although TAD-like domain structures are highly dynamic between cells, we found that they are not randomly distributed in the cell population, implying the existence of tight regulation on these domains at a single-cell level. Finally, the insulation property of TAD-like domain boundaries was also found to have a major effect on the epigenetic landscape in individual cells.

## Results

### A novel TAD detector (deTOKI) for ultra-low-resolution Hi-C data

**Fig. 1:**
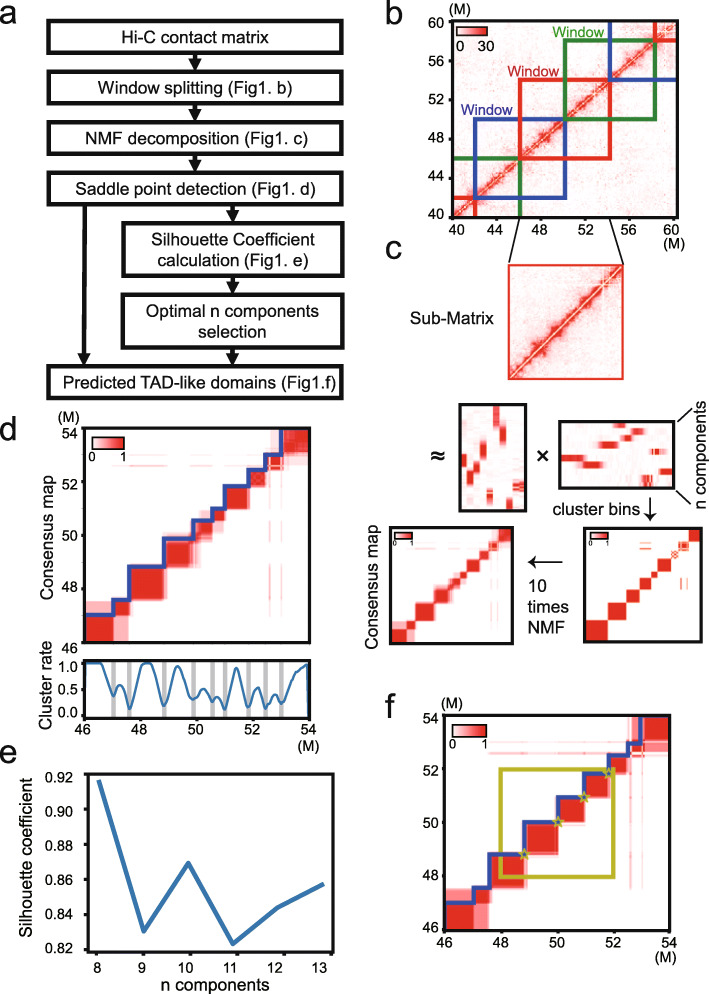
Workflow of deTOKI. **a** The flow chart of deTOKI. The genome is split into 8 MB overlapping sliding windows. As an example, **b–f** shows the key steps for a window in chr19. The data are from IMR90 cells [14]. In **b**, each square in blue, red, and green represents a sliding window. In **c**, NMF was performed on the contact matrix of a window, and bins were clustered based on the factor matrix. After 10 rounds of NMF, a consensus map was generated (**d**), in which the detected clustering change points are shown in sawtooth, and the blue curve in the bottom panel represents the clustering rate of each bin. **e** The blue curve represents the silhouette coefficient corresponding to each alternative value of “n_components”. **f** The yellow square highlighted the middle 4-Mb region for which clustering change points (yellow stars) were reported as TAD-like domain boundaries

Using ultra-sparse Hi-C contact matrices, we developed a novel algorithm, named deTOKI, to detect TAD-like domain structures, a term we use hereinafter to avoid confusion. The deTOKI takes advantage of a key property of TADs, namely that its topology distribution is relatively consistent with respect to number and length between cell types [14]. Briefly, for any given genome segment, deTOKI applies non-negative matrix factorization (NMF) to decompose the Hi-C contact matrix into genome domains that may be spatially segregated in 3D space (Fig. 1a). Non-negative matrix factorization is an algorithm for decomposition of a non-negative matrix into a product of two non-negative matrices, in which the n_components represent the common dimensions between the two decomposed matrices. As the n_components normally are substantially smaller than the dimensions of the origin matrix, NMF is a commonly used algorithm to perform dimension reduction [46]. To speed up the algorithm, deTOKI divides the chromosomes into 8-Mb sliding windows, overlapping each 4 Mb, and the clusters from the second Mb to the sixth MB of each window are reported as the predictions (“Methods,” Fig. 1, Additional file 1: Supplemental Note, Additional file 2: Fig. S1-2). The alternative local optimal solutions in the structure ensemble are achieved by summarizing deTOKI’s predictions with multiple random initiations (see “Methods”).

To assess deTOKI’s performance, we focused on two major characteristics of chromatin architecture at the single-cell level, i.e., data sparsity and cell-to-cell variations, and assessed them with downsampled experimental data and with simulated data, respectively. All analyses in this work were performed with binsize = 40 kb, unless otherwise mentioned.

### deTOKI worked well in downsampled bulk Hi-C at the single-cell level

**Fig. 2:**
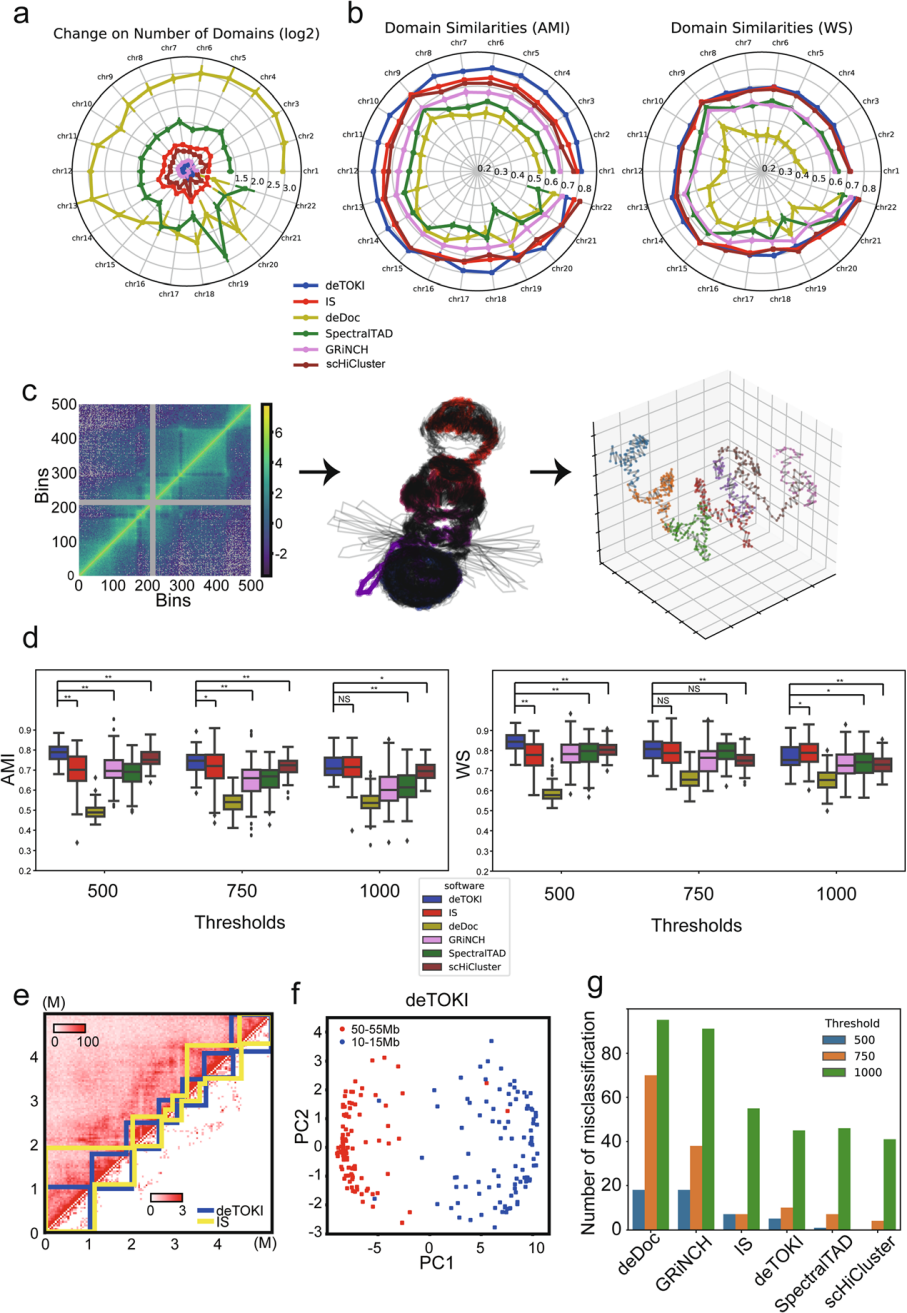
Comparison of TAD callers on downsampled and simulated single-cell Hi-C based on data from IMR90 [14]. Panels **a** and **b** show the average results of 20 independent downsamplings in each chromosome. **a** The (log2) change in the number of predicted TAD-like domains. **b** The similarity of TAD-like domains, as inferred by AMI and WS, between the raw data and the downsampled data. **c** Workflow of the single-cell Hi-C simulation. From left to right, the panels represent the normalized Hi-C contact matrix of chr18:50–55 Mb for GM12878 ensemble Hi-C from Rao’s data [18], an ensemble of 100 modeled 3D structures of this region, and the 3D structure modeled from the simulated ensemble Hi-C from model #100. Each dot in the right panel represents a 10 kb-length particle, and the dots with same color belong to the same predicted TAD-like domain ensemble. **d** Similarities of predicted single-cell TAD-like domains between different thresholds and predictors. **e** An example of the simulated data. The upper and lower parts of the heatmap represent the simulated reference and single-cell Hi-C data from model #13, *D* = 500. Predicted TADs are shown in sawtooth. AMIs between TAD-like domains predicted by deTOKI and IS on the two datasets are 0.873 and 0.660, respectively. **f** Classification based on deTOKI-predicted TAD-like domains of models on chr18:50–55 Mb and chr18:10–15 Mb, mimicking two single cells. Each dot represents a model, *D* = 500. **g** Number of misclassifications, using predicted TAD-like domains. *P < 0.05, **P < 0.001, NS: not significant, two-sided Mann-Whitney *U* test

To assess the performance of deTOKI under the condition of sparse input, we mainly compared it with two publically available algorithms, i.e., insulation score (IS) [30] and deDoc [42]. These two methods were chosen because they were judged to be the most robust methods with sparse data in our previous comprehensive assessments of TAD predictors [43]*.* In addition, we also compared it with recently published algorithms designed for sparse data, including SpectralTAD [47], GRiNCH [48], and scHiCluster [49]. These algorithms employ the data imputation method on single-cell Hi-C data and predict domains by TopDom. Sparsity was defined as the proportion of entries in the Hi-C matrix that have value zero after excluding the unmappable genome regions, e.g., centromeres, for a given chromosome. The assessment was done for all chromosomes in 40-kb bins and was downsampled at the rate of 1/800 from the high-resolution Hi-C data [14]. The downsampled dataset consisted of about 0.44 M contacts, mimicking the sequencing depths of public scHi-C datasets, e.g., the median of the data generated by Flyamer and colleagues (hereafter termed Flyamer’s data [50]) was 0.339 M (Fig. 2a, b).

The deTOKI outperformed the other tools in the following two respects. First, compared to the other tools, the number of TAD-like domains predicted by deTOKI and GRiNCH was little affected by data sparsity (Fig. 2a and Additional file 2: Fig. S3b). Taking chr10 as an example, the largest absolute log2 fold changes (|log2FC|) in the number of predicted TAD-like domains among the downsampled datasets was 0.26 for GRiNCH and deTOKI, while it was 0.51, 0.80, 1.38, and 2.40 for scHiCluster, IS, SpectralTAD, and deDoc, respectively (Fig. 2a). Second, on single-cell data, deTOKI predicted TAD-like domains more accurately than all other predictors. We took the TADs identified with the full data as the gold standard and quantified the accuracy of predictions by the similarity to the gold standard. Two similarity indexes, i.e., adjusted mutual information (AMI) [51] and weighted similarity (WS) [42], were employed. The deTOKI values had higher similarity than all of the other algorithms for both indexes in most chromosomes, i.e., in 19 and 16 out of 22 chromosomes for AMI and WS, respectively (Fig. 2b). We also employed two different indexes, BP score (BP) [52] and variation of information (VI) [38]. Although IS performed best with the two indexes (Additional file 2: Fig. S3a), deTOKI performed comparably well in all chromosomes (median BP = 0.49 and 0.51; median VI = 1.58 and 1.61, for IS and deTOKI, respectively).

Moreover, when we performed an additional assessment with binsizes 20 kb and 80 kb, deTOKI performed equally well with binsize 40 kb, as we described above, and better than the other tools (Additional file 2: Fig. S3b-c). Finally, marks of the characteristic structural protein CTCF, or histones, were found to be enriched in deTOKI-predicted TAD-like domain boundaries. Compared to the genomic background, ChIP-seq peaks of H3K4me3, H3K36me3, and CTCF were enriched at the TAD-like domain boundary regions predicted by deTOKI, IS, scHiCluster, and deDoc, while such enrichment was barely seen in those boundary regions predicted by GRiNCH and SpectralTAD (Additional file 2: Fig. S3d). These observations further supported the accuracy of the predictions. Taken together, our assessments suggest that deTOKI can stably and accurately predict TAD-like domains with ultra-low-resolution (i.e., single-cell level) Hi-C data.

### deTOKI worked well with simulated single-cell Hi-C data

To mimic cell-to-cell variation, we simulated a single-cell Hi-C experiment. The simulated data were generated according to the following protocol. First, we simulated chromosome structures for single cells. By applying a widely used 3D structure modeling tool known as IMP on the bulk Hi-C data, we modeled a 3D chromosome structure ensemble containing about 100 physical chromosome structure models such that each model represented a single cell (Fig. 2c) [53]. To simplify the simulation, we assumed that each modeled structure in the ensemble would be evenly distributed within the cell population. We randomly chose a 5-Mb-long genome region, i.e., chr18:50–55 Mb, as an example. To generate single-cell Hi-C data from the physical 3D model, we defined the Hi-C contacts as pairs of genome loci with a Euclidean distance less than a threshold (*D*) in the model. Hi-C reads were then sampled from the contacted genomic loci by a binomial distribution (see “Methods”). In this work, we tested three threshold *D*s, i.e., 500, 750, and 1000, representing 20%, 40%, and 60% quantiles, respectively, in the distribution of distances among all genomic loci pairs in the physical model (Additional file 2: Fig. S3g). To define the true domains (reference) in the given single cell, a sufficient number of reads were sampled from the physical model with the sampling probability function of a pair of interacting genome loci being inversely proportional to their Euclidean distance (see “Methods”). The expected number of reads sequenced from the loci was calculated by normalizing all weights, i.e., contact probabilities, in a genome-wide manner, and the Hi-C reads were then sampled from those genome loci by Poisson distribution.

The deTOKI can accurately predict domain structures in simulated single-cell Hi-C data. With the method described above, we simulated the structures of a 5-MB region in 100 single cells and generated about 1000 and 0.35 M Hi-C contacts for each single cell and the reference Hi-C, respectively. We compared the accuracies (i.e., AMI and WS) of the predictions of the predictors, and when estimated by AMI, we found that deTOKI had significantly higher accuracy than the other tools (Mann-Whitney *U* test, *P* < 0.001) for D = 500 and 750. With D = 1000, IS had the best performance (Fig. 2d); however, the median values of AMI and WS were similar for IS and deTOKI (AMI median = 0.715 and 0.708; WS median = 0.789 and 0.753, respectively). We also employed BP and VI to measure the differences between the predicted domains and the reference. deTOKI and IS also performed best with these two indexes (Additional file 2: Fig. S3f). This pattern was also seen in an additional randomly selected genome region (chr18:10–15 Mb, Additional file 2: Fig. S3f and h). For example, in model #13 and with D = 500, deTOKI-identified domains in simulated single cells matched very well with the associated reference Hi-C, while several major domains were mislabeled using the IS predictor (Fig. 2e).

Single cells could also be accurately classified by deTOKI-predicted domains. As an example, we took the two 5-Mb regions of chr11 to represent two types of cells since their separation by 40 Mb on the chromosome would result in few connections. For the 100 simulated models of the two regions, representing two cell types, and using WS as distance, deTOKI-predicted domains had better classification power for distinguishing the two cell types than all other tools, except SpectralTAD and scHiCluster (Fig. 2f). If we run deTOKI at imputed data from scHiCluster, we can get the best classification (Additional file 2: Fig. S10d). Furthermore, the total number of misclassified cells of deTOKI was lower than that of IS (Fig. 2g). The success of deTOKI as predictor on the simulated data encouraged us to further assess if the tool would work equally well on experimental single-cell Hi-C data.

### deTOKI predicts TAD-like domains with experimental scHi-C data

Next, we compared predictions with three experimental scHi-C datasets, hereinafter denoted as Flyamer’s, Tan’s, and Li’s datasets (Additional file 3-5: Table S1-3) [50, 54, 55]. We only compared deTOKI with IS, scHiCluster, and deDoc as the latter three were shown to perform relatively well with the simulated sparse data above. We found deTOKI’s predictions to be both more accurate and more stable than those of the three other tools.

**Fig. 3:**
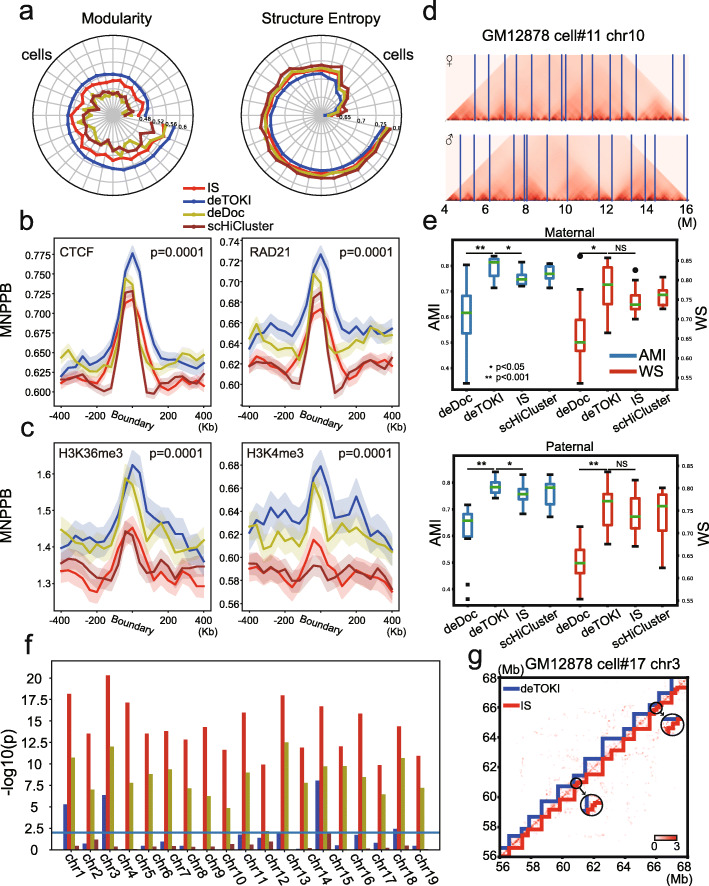
Comparison of TAD callers with Tan’s data [54]. **a** The radar plot shows the modularity and structure entropy of predicted TAD-like domains in chr1 in 32 cells (15 GM12878 cells and 17 PBMC cells). The cells were ordered by the modularity and structure entropy of deTOKI’s predictions. **b,c** Genome-wide distribution of ChIP-seq peaks of structural proteins (CTCF and RAD21) and histone marks (H3K4me3 and H3K36me3) flanking the single-cell TAD-like domain boundaries for the 16 GM12878 cells are shown in **b** and **c**, respectively. The shadows represent 95% confidential intervals, as calculated by bootstrap. The *y*-axis represents the mean number of peaks per bin with the same distance to the predicted TAD-like domain boundaries (MNPPB). The enrichment *p* values are calculated by the permutation test (*n* = 10,000). **d** deTOKI-predicted TAD-like domain boundaries match the boundaries predicted by 3D modeling [54]. The example shows the original matrix of radii of gyration for chr10, cell #11 of GM12878. The deTOKI-predicted allelic TAD-like domain boundaries are marked with vertical blue lines. **e** The AMI and WS between the 3D-modeled hierarchical TAD-like domains (as defined in [54]) and predicted allelic single-cell TAD-like domains in GM12878. **f** Significance levels of Pearson’s correlation coefficients between the number of contacts and the number of predicted TAD-like domains by deTOKI, deDoc, IS, and scHiCluster in each chromosome of 150 mESCs [55]. The threshold “*P* value = 0.01” is indicated by the horizontal blue line. **g** An example of mini-TAD-like domains predicted by deTOKI and IS. The mini-TAD-like domains in the circles are zoomed in as embedded sub-plots. The color codes for the four TAD predictors are all identical to those in **a**. *P < 0.05, **P < 0.001, NS: not significant, two-sided Wilcoxon rank-sum test

First, deTOKI predicted TAD-like domains with higher modularity and lower structure entropy. The modularity and structure entropy of a network have previously been used to infer the topological properties of TADs from the Hi-C contact matrix [41, 42]. A better defined TAD set is expected to have smaller structure entropy [42] and larger modularity [41]. With Tan’s and Flyamer’s datasets, deTOKI predicted TAD-like domains with lower structure entropies and higher modularities than those of TAD-like domains predicted by IS, scHiCluster, or deDoc (Fig. 3a, Additional file 2: Fig. S4a). For example, when we compared the predictions of IS, scHiCluster, and deDoc in Tan’s data against deTOKI-predicted TAD-like domains, we found that the TAD-like domains in chr1 had higher modularity and lower structure entropy in all cells, respectively (Fig. 3a). In Li’s data, deTOKI also performed best of the four predictors, having the highest modularity and lowest structure entropy in 77 and 73 cells, respectively (Additional file 2: Fig. S4a).

Second, the structural proteins and histone marks were more enriched at the deTOKI-predicted TAD-like domain boundaries in real single-cell data. By aggregating the ChIP-seq signals at the predicted TAD-like domain boundaries in all single GM12878 cells, we found that the deTOKI-predicted TAD-like domain boundaries had higher enrichment of CTCF and Rad21(cohesin) compared to IS, scHiCluster, and deDoc (Fig. 3b). This was also true for H3K36me3 and H3K4me3, the two histone marks previously reported to be enriched in the ensemble TAD boundaries (Fig. 3c) [14].

Third, deTOKI-predicted single-cell TAD-like domains were more consistent with the modeled physical structures. Xie and colleagues modeled the physical structure of the haploid chromosomes of single GM12878 cells at 10 kb resolution and proposed an algorithm to infer the chromosome domains from the hierarchical physical structure [54]. Using this haploid physical model and algorithm, we inferred the chromosome domains in a randomly chosen genome region (chr10:4–16 M, see “Methods”). Compared with the deTOKI-predicted haploid single-cell TAD-like domains, we found that deTOKI-predicted single-cell TAD-like domain boundaries matched the 3D modeling very well (Fig. 3d). Using AMI and WS as the indexes, we compared the 3D-modeled hierarchical domains with the TAD-like domains predicted by the three predictors [54] (Fig. 3e). In both maternal and paternal chromosomes, the AMIs of deTOKI’s prediction were significantly higher than those predicted by IS (*P* = 0.04 and 0.02, respectively, two-sided Wilcoxon rank-sum test). The WS of deTOKI’s prediction was also higher than that predicted by IS (*P* = 0.1 and 0.35, respectively). As the total number of cells and TAD-like domains in this comparison was small, i.e., about 15–25 TAD-like domains, we think the significance of the WS was acceptable.

Last, deTOKI exhibited a more stable performance compared to IS. Using chr1 in PBMC cell #14 as an example, we performed 20 rounds of 50% downsampling on the single-cell Hi-C reads and predicted TAD-like domains from the downsampled data. Overall, the predictions of both predictors remained largely intact. For example, the distribution of TAD-like domain lengths remained similar between the full and the 50% downsampled data (Additional file 2: Fig. S4b-c). In terms of AMI and WS, deTOKI, IS, and scHiCluster performed equally well, i.e., AMI = 0.90, WS = 0.85 and AMI = 0.90, WS = 0.87 and AMI = 0.89, WS = 0.87, respectively (Additional file 2: Fig. S4d-e). The AMI and WS of deDoc were 0.80 and 0.71, which are lower values than those of deTOKI. However, deTOKI outperformed IS in two respects. First, the number of deTOKI-predicted TAD-like domains relied much less on reads coverage compared to IS. With Li’s data, the number of IS-predicted TAD-like domains was strongly correlated with reads coverage on all chromosomes, while for deTOKI, only a moderate correlation in this respect was found on five out of nineteen chromosomes (Fig. 3f). Second, IS predicted more questionable mini-TAD-like domains, i.e., length < 100 kb. Mini-TAD-like domains were typically found in the ultra-sparse region, depending on reads coverage. Within all the TAD-like domains, 0.29% and 9.81% were considered as mini-TAD-like domains, as predicted by deTOKI and IS (Fig. 3g and Additional file 2: Fig. S4f-g), in the single cell, respectively.

Taken together, our assessment suggests that deTOKI works well with experimental single-cell Hi-C data.

### The improvement of deTOKI with data imputation

Data imputation is a commonly used strategy when handling single-cell data, e.g., scHiCluster [49] and Higashi [56]. We assessed the performance of deTOKI running on imputed data by comparing deTOKI with data imputation and Higashi (Additional file 1: Supplemental Note, Additional file 2: Fig. S10). We found that deTOKI and Higashi performed in a similar manner and that deTOKI could be further improved by data imputation, e.g., scHiCluster. Considering the substantial CPU time required by Higashi (about 100-fold more CPU time than that required by deTOKI or scHiCluster; see Additional file 6: Table S4), deTOKI is more efficient on TAD-like domain identification with single-cell Hi-C data than Higashi.

### TAD-like domain structure is highly dynamic at the single-cell level

**Fig. 4:**
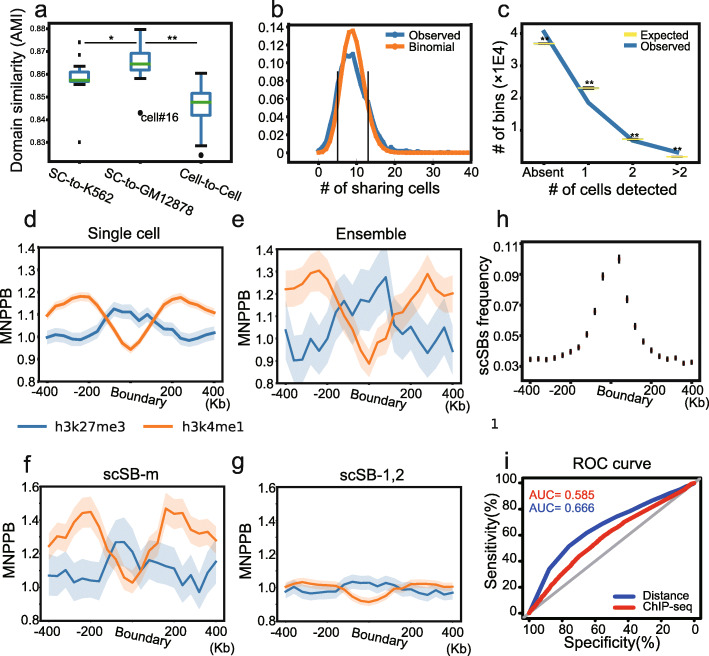
The dynamics of TAD-like domains in single cells. **a** The cell-to-cell and cell-to-ensemble similarity of the deTOKI-predicted TAD-like domains. The single-cell data were from GM12878 [54] and compared to the ensemble in GM12878 and K562. Cell #16 was marked as it was in M/G1 phase. **b** The distribution of ensemble TAD boundaries over single cells. The control was set as a binomial distribution under the hypothesis that every ensemble TAD boundary has identical potential of being a single-cell TAD-like domain boundary. The vertical black lines marked 13 and 5 indicate the thresholds for the over- and under-represented boundaries in the cell population, respectively. **c** The distribution of bins among the classes “scSB-1,” “scSB-2,” “scSB-m,” and “absent.” The permutated control is shown in the boxplots. The distribution of H3K27me3 and H3K4me1 histone marks flanking the deTOKI-predicted single-cell-specific ensemble, scSB-m, scSB-1, and scSB-2 TAD-like domain boundaries are shown in **d–g**, respectively**.** The *y*-axis of panels **d–g** represents the mean number of peaks per bin with the same distance to the predicted TAD-like domain boundaries normalized by the average in the whole genome (MNPPB). The shadows represent 95% confidential intervals, as calculated by bootstrap. **h** The distribution of scSBs flanking the ensemble TAD boundaries. **i** The ROC curves of classification between scSB-1, scSB-2, and scSB-m based on either ChIP-seq peaks or the distance to the nearest ensemble boundaries. *P < 0.05, **P < 0.001, NS: not significant, two-sided Wilcoxon rank-sum test

Using AMI as the index, we investigated the cell-to-ensemble and cell-to-cell similarity of TAD-like domains. Therefore, when we compared cell-to-ensemble and cell-to-cell AMIs in Tan’s data (GM12878 single cells, chr1), we found TAD-like domains in single cells to be more similar to ensemble than individual cells based on the comparison of AMIs (Fig. 4a, Additional file 2: Fig. S5a-b). In other words, cell-to-ensemble AMIs were significantly higher than cell-to-cell AMIs in all three scHi-C datasets tested. Intriguingly, the average cell-to-cell AMI is even smaller than the cell-to-ensemble AMI of another cell type, e.g., single cells of GM12878 *vs*. ensemble of K562 (Fig. 4a). For example, the AMI of cell (GM12878)-to-ensemble (K562) and cell-to-cell (GM12878) AMI are 0.858 and 0.848, respectively (two-sided Wilcoxon rank-sum test, *P* < 0.001, Fig. 4a). Thus, our data suggested that the TAD-like domain structure in single cells is quite dynamic, even bigger than inter-cell-type variation. The pattern we showed above is not specific to GM12878, as it can also be seen in the other two tested single-cell Hi-C datasets (Additional file 2: Fig. S5a-b). We note that the average AMI between single cells of GM12878 and ensemble of GM12878 is significantly higher than that of ensemble of K562 (Fig. 4a). We tested the assumption that TAD-like domain structure carries information for cell identity in the section subtitled “TAD-like domain structure carries information for cell identity” below.

Considering that TADs are conserved between cell types [14], two possible scenarios may explain the above high-level dynamics of the TAD-like domain structure in single cells. First, each individual cell employs a subset of the ensemble TADs. Second, each cell has a certain number of additional cell-specific TAD-like domains. To test which scenario is the more prevalent in the cell populations tested, we roughly defined three types of variations between TAD-like domains, namely, merge, split, and shift (see Additional file 2: Fig. S5c and “Methods”), where merge does not generate novel TAD-like domain boundaries, while split and shift do. Using chr1 as an example, we found, on average, 31.8%, 22.3%, and 26.6% of merge, split, and shift TAD-like domains, respectively (Additional file 2: Fig. S5c-e), implying that a notable number of TAD-like domain boundaries do not appear in the ensemble TAD structures. We term such boundaries as single-cell-specific boundaries (scSB). In the next two sections, we will sequentially discuss the dynamics of ensemble boundaries and scSBs.

### Unnested ensemble TADs were frequently seen in single GM12878 cells.

We asked whether the ensemble TAD boundaries were purely randomly distributed in single cells. A simple assumption for this randomness would be that the distribution of the ensemble TAD boundaries is binomial in the cell population. To examine this assumption, we chose Li’s data as an example and modeled the distribution with a binomial *B* (150,0.06) [55], where 150 is the number of cells and 0.06 is the average frequency with which an ensemble TAD boundary appears in a single cell. We found that 453 and 452 boundaries (out of 2602) appeared in more than 12 and in less than 6 cells, respectively (Fig. 4b). Those numbers significantly deviate from the expectation of binomial null hypothesis (*P* < 0.001). This finding suggests that a group of ensemble TAD boundaries, termed as popular boundaries, occurs more frequently in the cells, while another group of boundaries, termed as unpopular boundaries, tends to be specific to a subpopulation of the cells. GO analysis showed that genes on the popular boundaries are enriched for terms related to cellular responses to DNA damage stimuli (*P* = 2.21E−3), while genes on the unpopular boundaries are enriched for terms related to negative regulation of cell-matrix adhesion (*P* = 1.52E−4, Additional file 2: Fig. S6d-f). This result further supported the assumption of a nonrandom distribution of ensemble TAD boundaries in single cells.

Both nested and unnested TAD boundaries were found in the ensemble [26]. We asked how these two types of boundaries are distributed in single cells. We chose chr1 in the GM12878 cells Hi-C data (termed hereinafter as Rao’s data [18]) as an example. We defined the nested and unnested boundaries and compartment domains, as previously described (see “Methods” [26]). Interestingly, by comparing the number of cells that carry such boundaries, we found that unnested boundaries were significantly enriched in single cells. In the 15 single cells, the 20 nested ensemble TAD boundaries appeared 14 times, while the 20 unnested ensemble boundaries appeared 44 times, being significantly more common than nested ones (*P* value = 0.003, two-sided Wilcoxon rank-sum test, Additional file 2: Fig. S6a-c). Taken together, our analysis suggested that ensemble TADs are dynamic in nature and that unnested ensemble TAD boundaries are more frequently chosen in single GM12878 cells.

### Single-cell-specific TAD-like domain boundaries may adhere to the ensemble boundaries

The scSBs may not result entirely from stochastic fluctuation. First, we identified a large number of single-cell-specific boundaries (scSB) using deTOKI. About 89.3% of TAD-like domain boundaries in single cells were not found in the ensemble if we defined two boundaries as identical when they were in the same bin. Those scSBs were less likely to result from coverage bias, as strong correlation between the scSBs and read coverage was rarely seen (Cor = 0.296, *P* = 0.284). Because of data sparsity, not all chromosomes found reads in every cell. For this analysis, we therefore only looked at the largest chromosome (chr1) for which reads were found in most cells. The following analysis was performed on the whole genome. Second, the distribution of scSBs in the cell population is not random. We grouped all scSBs into 3 classes by the number of cells that carry these scSBs (number = 1, =2, > 2, denoted as scSB-1, scSB-2, and scSB-m, respectively). Compared to permutated controls, far more scSBs in the scSB-m class were found and far fewer scSBs in the scSB-1 and scSB-2 classes (*P* < 1E−4, Fig. 4c). Moreover, bins not taken as boundaries for any cells in our data were also more prevalent than permutated controls (“Absent” in Fig. 4c). These results imply that the bins are either deficient or relatively prevalent, i.e., either with too few or too many cells to function as domain boundaries, respectively.

Third, scSBs have characteristic histone marks. We mapped all histone marks (except for H3K4me3 and H3k36me3) that have publically available ChIP-seq data for GM12878 cells in ENCODE. The distribution of histone marks showed either enrichment or depletion at the scSBs, similar to the ensemble TAD boundaries. For example, H3K27me3 and H3K4me1 were enriched and depleted around the boundaries in single cells, respectively (Fig. 4d, e; Additional file 2: Fig. S7a-b). However, this pattern fluctuated with larger variation around ensemble boundaries (Additional file 2: Fig. S7d). We also observed a similar enrichment in IS- and deDoc-identified TAD-like domain boundaries (Additional file 2: Fig. S7a-b). This enrichment of H3K27me3 was higher in scSB-m than that in scSB-1 and scSB-2 (Fig. 4f, g, Additional file 2: Fig. S7e). Indeed, more ChIP-seq peaks represented histone marks in scSB-m (Additional file 2: Fig. S7c and f). This line of evidence suggests additional constraint above the stochastic random walk.

To investigate plausible constraints on the scSBs, we compared them with the ensemble boundaries in chr1. We found a strong association between the two classes. First, 7.89% of the scSBs in GM12878 can be found in K562 ensembles, which means that at least some of the GM12878 single-cell-specific boundaries are likely to be insulative in the K562 ensemble. Second, the bins that carry scSBs tend to be close to ensemble TAD boundaries. 18.9% of scSBs are located within an 80 kb (± 40 kb) region flanking the ensemble boundaries (Fig. 4h), and the average distance to the nearest ensemble boundaries from scSB-m is significantly smaller than that from both scSB-1 and scSB-2 (Additional file 2: Fig. S7g). Last, we built a simple logistic regression model to distinguish scSB-1 and scSB-2 from scSB-m using the number of ChIP-seq peaks as features, and we found 5 features, including CTCF, H3K4me1, H3K4me2, H3K9ac, and H3K36me3, that were most relevant in this respect (Additional file 2: Fig. S7h). However, the AUC (0.585) was much lower than the AUC (0.666) of a model that directly used the shortest distance to an ensemble TAD boundary as the feature (Fig. 4i), suggesting that distance is the most important factor restricting the biogenesis of scSB. The importance of distance suggests that genesis of scSBs may not be completely random, but rather tends to fall within certain restricted regions common to all, or most, human cells, and which is, at least to some extent, represented by the ensemble boundaries.

Altogether, our analysis indicates that a large amount of cell-to-cell variations in the TAD-like domain structure, the prevalence of cell-specific domain boundaries in cells, and a large portion of the single-cell-specific boundaries may not purely result from stochastic fluctuation in single cells.

### The TAD-like domain structure carries information for cell identity

**Fig. 5:**
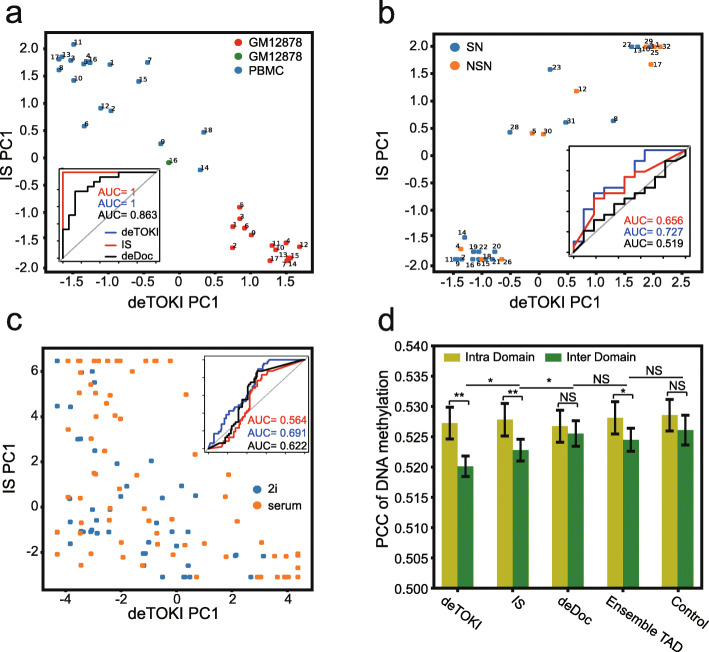
The deTOKI-predicted single-cell TAD-like domains characterize cell identity. The classification of single cells based on predicted TAD-like domain boundaries in Tan’s, Li’s, and Flyamer’s datasets are shown in **a–c**, respectively. Each dot represents a cell. The *x-* and *y-*axis represent the PC1 calculated by deTOKI and IS, respectively. The embedded plots show the AUC of the classification by each program. Cell #16 is colored in green in panel **a,** as it was in M/G1 phase. **d** The correlation of DNA methylation between bin pairs. The “inter-TAD-like domain” indicates that the two bins are separated by a predicted TAD-like domain boundary, while the “intra-TAD-like domain” indicates that the two bins belong to the same predicted TAD-like domain. Only bins fulfilling the criteria of (1) being members of pairs separated by 240 kb and (2) having reads containing a total of 2 or more CpGs, were included in this plot. *P < 0.05, **P < 0.001, NS: not significant, Fisher’s *z*-test [68]

Previously, Tan et al. showed that cell types can be classified using single-cell Hi-C data combined with sequence features of the reads [54]. Now we ask whether the TAD-like domain structure alone can be used to classify single cells. Using WS as the similarity index for all three single-cell Hi-C datasets, we found that single cells could be correctly classified by the TAD-like domain structure alone. Tan’s dataset [54] consists of two cell types, GM12878 and PBMC. Both deTOKI and IS can completely distinguish the two cell types using the predicted TAD-like domain as a feature (AUC = 1.0, 1.0, and 0.863, for deTOKI, IS and deDoc, respectively, Fig. 5a). Flyamer’s dataset [50] consists of non-surrounded nucleolus (NSN) and surrounded nucleolus (SN) oocyte cell types, representing transcriptionally active immature and inactive mature oocytes, respectively [50]. The deTOKI could distinguish these two cell types much better than either IS or deDoc (AUC = 0.73, 0.66 and 0.52 for deTOKI, IS, and deDoc, respectively, Fig. 5b). Flyamer’s dataset also consists of zygote-mats and oocytes. The deTOKI distinguished these better as well (AUC = 1.0, 1.0, and 0.89 for deTOKI, IS, and deDoc, respectively, Additional file 2: Fig. S8e). In Li’s data [55], the Methyl-HiC data consists of 150 single cells cultured in two different media: 2i and serum. We found that the TAD-like domains predicted by deTOKI could also better distinguish cells with different growing conditions than could IS and deDoc (AUC = 0.691, 0.564, and 0.622 for deTOKI, IS, and deDoc, respectively, Fig. 5c). The classification of cells in different growth media may not be trivial, as the GO analysis showed that the genes on serum-specific TAD-like domain boundaries were enriched for the term “DNA methylation on cytosine” (Additional file 2: Fig. S8a-b, *P* = 7.28E−4), which is consistent with the fact that serum-cultured mESC has a higher DNA methylation rate [55]. The serum-specific TAD-like domain boundaries were also enriched for the gene regulation-related GO terms, e.g., positive regulation of gene expression and epigenetic features (*P* = 8.20E−4), which agrees with the fact that serum-cultured mESCs have more heterogeneous transcriptional activity than cells cultured in 2i [57–59]. Further, epigenetic features were distinguished between the serum- and 2i-specific TAD-like domain boundaries (Additional file 2: Fig. S8c). Altogether, deTOKI predicted TAD-like domains in single cells carrying reliable information about cell identity.

### The DNA methylation pattern is highly correlated between TAD-like domain boundaries at the single-cell level

It has been suggested that spatially approximated genome loci are prone to share similar epigenetic patterns [60]. We thus asked if this feature could also be seen in single cells. In this case, we would expect to see lower correlations in DNA methylation between the inter-TAD-like domain bins than that between intra-TAD-like domain bins in the single cells. To test this speculation, we looked at Li’s data [55]. First, at the ensemble TAD level, the genome loci flanking the strongly insulated TAD boundaries have lower correlations on DNA methylation than those flanking the weakly insulated TAD boundaries (Additional file 2: Fig. S8d). The ensemble boundaries were classified into strong and weak groups with an identical number according to the insulation scores. The ensemble TAD boundaries of mESC were downloaded from the work of Dixon and colleagues [14], and the insulation scores at those boundaries were calculated by the pooled contact matrix of Li’s data. The average PCCs were 0.546 and 0.490 for weak and strong boundaries, respectively, and this correlation could also be seen if the boundaries were classified into more groups (Additional file 2: Fig. S8d). Next, we examined the inter-TAD-like domain and intra-TAD-like domain PCCs of DNA methylation level in the single cells. Indeed, when the TAD-like domains were defined by deTOKI or IS (Fig. 5d), the intra-domain PCCs were significantly larger than the inter-TAD-like domain PCCs, while when the TAD-like domains were defined by deDoc or the shuffled control, little difference was noted. The PCCs of inter-domain bins from deTOKI-predicted TAD-like domains were significantly lower than those from IS-predicted TAD-like domains (PCC = 0.520 *vs*. 0.523 for deTOKI and IS, respectively, *p* = 0.003), implying that the boundaries predicted by deTOKI are more spatially insulated in single cells. Although the average PCC between inter-domain bins was relatively low, it remains notable. We speculate that this might be caused by the existence of weak TAD boundaries, as discussed above. Together, our analysis suggested that spatially approximate chromatin loci are prone to carry similar epigenetic features and that the dynamic nature of TAD-like domain structures at the single-cell level has notable consequences for the ensemble of the epigenetic landscape.

## Discussion

In present work, we have developed a TAD-like domain identification algorithm that can work on sparse data at the single-cell level. We assessed the accuracy and robustness of deTOKI in downsampled, simulated, and experimental single-cell Hi-C data, and we compared deTOKI to the two best-performing tools on sparse data, IS, and deDoc [43]. The assessment showed that deTOKI not only outperformed IS and deDoc, but also reliably predicted TAD-like domains in experimental single-cell Hi-C data and is thus the first published tool with such capacity.

We took advantage of NMF on handling sparse data for decomposition of the Hi-C contact matrix. NMF has been widely used in single-cell data analysis, e.g., coupled NMF [45]. The boundaries defined by deTOKI were the optimal saddle points, which are also the genome loci that insulate chromatin interactions. The combination of NMF and insulation detection enabled deTOKI to achieve reliable TAD-like domain prediction on sparse data.

Future deTOKI work will involve the following features. First, we will improve sensitivity in the contact desert regions. New experimental technologies for higher data coverage have been able to reach the contact desert region [54], but algorithms can still be improved. We noticed that several tools have been uploaded to the preprint severs, e.g., Higashi [56] and 3DVI [61], to integrate embedding and data imputation for single-cell Hi-C data and, therefore, improve data quality. To fully address the issue of contact desert region, the approaches that integrate sensitive TAD detectors, e.g., deTOKI, we introduced here, data imputation and new experimental technologies may be necessary. Second, introduction of a better assessment for TAD-like domain reliability would be extremely useful when the detection probes the deep contact desert regions. Finally, deTOKI needs to gain some speed. Although the current running speed of deTOKI is acceptable, it is slower than deDoc. Parallelization is one way to improve the speed, as deTOKI works on split genome fragments. However, we sought to optimize the algorithm so that access to a supercomputer is not necessary to scan the whole genome.

With the ability of probing TAD-like domain structures in single cells, we examined the dynamics of the domain boundaries. Three novel features were revealed. First, although cell-to-cell variation is large, most single-cell TAD-like domain boundaries adhered to the ensemble consensus. Since only a small fraction of boundaries in the ensemble can be detected in each single cell, the dynamics of TAD-like domains is likely to be high. However, since most scSBs adhered to the ensemble consensus, this may indicate the existence of subpopulations in the isogenic cell population. Whether the cells would constantly stay in one subpopulation or switch between subpopulations will be an interesting question to ask in future studies. Second, our data showed that TAD-like domain boundaries are prone to be unnested TAD boundaries, while little bias was noticed in compartment domain. This result may indicate that the biogenesis of TADs and compartment domain differ in principle. The last novel feature is the enrichment or depletion of certain histone marks at, or flanking, the scSBs (Fig. 4d–g, Additional file 2: Fig. S7d-e). As we do not have single-cell ChIP-seq data available, it would be interesting to ask if those histone marks can, indeed, be observed in single cells. If the answer is in the affirmative, then many as yet undiscovered properties of scSBs may be linked to the function of TAD-like domains in single cells. Preliminary GO analysis showed strong association between the enriched functional terms and cell identity (Additional file 2: Fig. S8), hinting at the profound functions the scSBs may have. To further reveal the mechanisms of 3D genome folding, the principle and function of the domain structure at the single-cell level will be key questions to ask. The deTOKI provides a basic tool for addressing those questions.

## Conclusions

We developed a new method, termed deTOKI, using NMF to decode TAD-like domain boundaries from ultra-sparse Hi-C data. The deTOKI not only outperformed IS and deDoc, but also reliably predicted TAD-like domains in experimental single-cell Hi-C data. By applying deTOKI to public experimental single-cell Hi-C data, we found that the domains adhere to the ensemble, even though the TAD-like domain structure is highly dynamic between the cells, suggesting their tight regulation. Finally, we found that the insulation property of the TAD-like domain boundaries also has a major effect on the epigenetic landscape in individual cells.

## Methods

### The simulation of single-cell and reference Hi-C

To simulate Hi-C, we constructed a 3D model using IMP with default settings at 10 kb resolution for any given 5 Mb genome region [53]. We simulated the reference and single-cell Hi-C data as follows [62].

Simulation of the reference Hi-C: For any two genome loci *i* and *j*, the weight was set as

*Weight(i, j) = 1/distance(i, j),*

where the distance was Euclidean. The chance of being sequenced in a Hi-C was then set as the normalized weights, and the expected read number was calculated by the chance times the total number of reads. The normalization was performed so that the total number of sequence reads was identical to that of widely used bulk Hi-C data [14], being the equivalent of 0.35 M reads per 5-Mb region. Hi-C reads were simulated by Poisson distribution with the expectation calculated above.

Simulation of the single-cell Hi-C. For any two genome loci *i* and *j*, the weight was set as

*Weight(i, j) = D-distance(i, j),*

where the distance was Euclidean, and *D* is the threshold. Only genome loci having a Euclidean distance less than D were considered to be contacting. The chance of being sequenced in a single-cell Hi-C was then set as the normalized weights, and the expected read number was calculated by the chance times the total number of reads. The normalization was performed so that the total number of sequenced reads was identical to Tan’s data [54], being equivalent to 1000 reads per 5-Mb region. Hi-C reads were simulated by Binomial distribution with the expectation just calculated. The 40 kb resolution Hi-C contact matrix was used for actual TAD-like domain detection. Thus, the simulated 10 kb resolution matrix was binned into 40 kb resolution.

### Processing of Hi-C data

Bulk Hi-C data were normalized by the ICE method [63], while we did not normalize single-cell Hi-C data owing to its sparse nature, and we also bypassed the normalization step on the downsampled data when its sparsity was comparable to that of single cells. In this work, we used a sampling rate of 1/800 at the single-cell level. All simulated and experimental data used in this study are summarized in Additional file 7: Table S5.

### deTOKI

We split the chromosomes into a series of overlapped sliding windows with length equals *L* (Mb). Thus, the neighbor windows overlapped each other by *L/2* Mb (Fig. 1b). We removed windows with fewer than 100 intra-window contacts. The default *L* is 8 Mb in deTOKI. The TAD-like domains were then predicted as follows.

1. The clustering of bins. In each 8-Mb window, we perform NMF on its contact sub-matrix by the function “sklearn.decomposition.NMF” of the scikit-learn package in Python [44], with “random” being the initialization setting (Fig. 1c). The parameter “n_components” represents the dimension of the factor matrices. The parameter “n_components” traverses an appropriate interval according to the average length of the TAD(s) and the length of the window. The suggested numbers for mammalian cells were 8, 9, …, 13. Bin *i* and bin *j* are clustered if the maximums in columns *i* and *j* of the coefficient matrix are in the same row.
2. Domain boundary detections. For each candidate of “n_componets” equal to *n*, we perform NMF *k* times in which the seed for random initialization, namely “random_state,” traverses within the interval [0, k-1]. The default *k* was 10, as we found little difference on the predicted TAD-like domains between *k = 10* and *50* for both single-cell and bulk Hi-C data (Additional file 2: Fig. S1a). We define a consensus map ***C***, ${C}_{i,j}=\frac{x}{k},i,j=1,2\dots 200,$where *x* denotes the number of NMFs that have bin *i* and bin *j* clustered together. Then, the cluster rate of any given bin *i* (*CR*_*i*_) was defined as the average value of all elements in the sub-square-matrix of ***C*** cornered at (*i, i*) with 11 bins along the matrix diagonal. *CR*_*i*_ is called the local minimum cluster rate if the following inequality is satisfied:

$$ \sum \limits_{j=i}^{i+2}{CR}_j-\sum \limits_{j=i-3}^{i-1}{CR}_j<0\le \sum \limits_{j=i+1}^{i+3}{CR}_j-\sum \limits_{j=i-2}^i{CR}_j. $$

The location and strength of the bins that have local minimum cluster rates were recorded. We assume the location set is {*i*_1_, *i*_2_, …, *i*_*m*_}. For any *t = 1…m*, the strength of ${bin}_{i_t}$ was defined as the local maximum *CR* minus ${CR}_{i_t}$.

$$ Strength\ of\ {bin}_{i_t}=\mathit{\max}\left\{{CR}_{i_{t-1}},{CR}_{i_{t-1}+1},\dots, {CR}_{i_{t+1}}\right\}-{CR}_{i_t}, $$

where *i*_*0*_ *= 1, i*_*m + 1*_ *= 200.* Thus, the TAD-like domain boundaries were defined as the strongest *n − 1* bins and the points that have a *CR* strength larger than 0.3 (Fig. 1d).

3. The silhouette coefficient calculation. The silhouette coefficient was introduced to provide an evaluation of clustering validity, and it is often used to select an “appropriate” number of clusters [64]. For each candidate of “n_componets,” we calculated the silhouette coefficient between the consensus map *C*_*i*, *j*_ and the TAD-like domain boundaries {*i*_1_, …*i*_*m*_}, as
  $$ {i}_0=1;{i}_{m+1}=201;{D}_{i,j}=1-{C}_{i,j} $$

$$ \mathrm{Silhouette}\ \mathrm{coefficient}=\frac{\sum_{k=0}^m{\sum}_{a={i}_k}^{i_{k+1}}\frac{mean_{1\le b<{i}_k\  or\ {i}_{k+1}\le b<201}{D}_{a,b}-{mean}_{i_k\le b<{i}_{k+1}}{D}_{a,b}}{\ \mathit{\max}\left({mean}_{1\le b<{i}_k\  or\ {i}_{k+1}\le b<201}{D}_{a,b},\kern0.5em {mean}_{i_k\le b<{i}_{k+1}}{D}_{a,b}\right)}}{200}. $$

The candidate *n* with the biggest silhouette coefficient was chosen, and its associated domain boundaries were considered as the final prediction (Fig. 1d, e).

4. The reported TADs. The deTOKI reports the clusters in the middle half of each window, i.e., the region from the *L/4* to *3 L/4* window, as the predictions.
5. Suggested parameter settings. For low-resolution Hi-C data, we recommend 8-Mb window and 40-kb binsize as the proper setting. Although the difference of predicted TAD-like domain between different binsize for both single-cell and bulk Hi-C data were found to be minor, they are adjustable as parameters in deTOKI (Additional file 2: Fig. S1b).
6. Running time. As deTOKI runs on each sliding window independently, the overall complexity can be written as *O(n)* where *n* represents the number of sliding windows for the whole genome. This complexity can also be evidenced by the almost linear correlation with the chromosome lengths (Additional file 2: Fig. S1c). Within each window, let the window size be *L* and the binsize be *B*, and we have the contact matrix size = *L/B.* Because the basic computing unit in deTOKI is NMF, which has the complexity of O(*ab*), where *a* and *b* represent the number of rows and columns in the input matrix, i.e., the contact matrix, the complexity for each NMF is *O((L/B)*^2^*)*. Together, the total computing complexity is O(n*(L/B)^2^*).* It took about 1 h to identify TAD-like domains in 40 kb resolution data of the whole genome of mm9 with Flyamer’s data [50]. The testing was performed in a computer with Intel(R) Xeon(R) CPU E5-2640 v3 @ 2.60 GHz with one core, and it could be as fast as finishing the same job in 6 min when using 16 cores (Additional file 2: Fig. S1c).

### Execution of other TAD predictors

Most of TAD predictors were executed with default parameters. We removed the mini-TADs predicted by deDoc, i.e., TADs shorter than 200 kb and 300 kb, in the simulated and experimental single-cell data, respectively. We calculated hierarchical domain and radii of gyration in the single-cell Hi-C data according to Tan et al. [54]. To properly compare hierarchical domains and TAD-like domains, we cut the hierarchical tree such that the number of domains and TAD-like domains were similar. The running time for each tool can be found in Additional file 6: Table S4.

### The similarity of two sets of TAD-like domains

Given two sets of domains, *T* = {*T*_1_, *T*_2_, …, *T*_*n*_} and *K* = {*K*_1_, *K*_2_, …, *K*_*m*_}, *N* is the length of contact matrix. We assess their similarity using adjusted mutual information (AMI) [51], weighted similarity (WS) [42], BP distance (BP) [52], and variation of information (VI) [38].

### Adjusted mutual information AMI (*T*, *K*)

Mutual information MI (*T, K*) was defined as

$$ \mathrm{MI}\left(T,K\right)=\sum \limits_{i=1}^n\sum \limits_{j=1}^mP\left(i,j\right)\mathit{\log}\left(\frac{P\left(i,j\right)}{P(i){P}^{\prime }(j)}\right), $$

where

$$ P\left(\mathrm{i}\right)=\raisebox{1ex}{$\left|{T}_i\right|$}\!\left/ \!\raisebox{-1ex}{$N$}\right.;{P}^{\prime }(j)=\raisebox{1ex}{$\left|{K}_j\right|$}\!\left/ \!\raisebox{-1ex}{$N$}\right.;P\left(i,j\right)=\raisebox{1ex}{$\left|{T}_i\bigcap {K}_j\right|$}\!\left/ \!\raisebox{-1ex}{$N$}\right.. $$

Then, the adjusted mutual information AMI (*T*, *K*) was defined as

$$ \mathrm{AMI}\left(T,K\right)=\frac{MI\left(T,K\right)-E\left\{ MI\left(T,K\right)\right\}}{\max \left\{H(T),H(K)\right\}-E\left\{ MI\left(T,K\right)\right\}}, $$

where *H* denotes the standard Shannon entropy, and *E* denotes expectation. AMI was calculated by the function adjusted_mutual_info_score in the Python module sklearn.metrics. In real calculation, all predicted TAD-like domains and intermediate windows of TAD-like domains are included in *T* and *K.*

### Weight similarity WS (*T*, *K*)

The weight similarity WS (*T*, *K*) was defined as

$$ \mathrm{WS}\left(T,K\right)=\frac{\sum \limits_{j=1}^m{S}_K^T(j)\ast \left|{K}_j\right|}{\sum_{j=1}^m\left|{K}_j\right|}, $$

where

$$ {S}_K^T(j)={\mathit{\max}}_{i=1}^n\left\{\frac{\left|{T}_i\bigcap {K}_j\right|}{\sqrt{\left|{T}_i\right|\ast \left|{K}_j\right|}}\right\}. $$

Because WS is an asymmetric index for similarity, we always put the predicted TAD-like domains from raw data in *T* and the TAD-like domains from downsampled data in *K*, while the intermediate windows of the domains were not included in either *T* or *K*.

### The enrichment of ChIP-seq peaks at the boundary region of domains

For any given resolution, e.g., 40 kb, a boundary region was represented by a vector of 21 entries, where the 1st to the 10th entries represent upstream 10 bins, the 12th to the 21st entries represent downstream 10 bins, and the 11th bin represents the middle point of the boundary. The value of each entry is the number of ChIP-seq peaks in each bin, and the middle entry is the total number of peaks in this boundary. These vectors are then summarized up to a total vector *v*_*i*_(*i* =  − 10, −9, …, 9, 10). We performed a permutation test for enrichment on the genome region of { *v*_−1_ ⋃ *v*_0_ ⋃ *v*_1_} and a Mann-Whitney *U* test between { *v*_0_} *and* { *v*_−10_ ⋃ *v*_10_} for the significance of the pattern. We define the MNPPB (mean number of peaks per bin) to reflect the enrichment of ChIP-seq peaks on the boundary of TAD-like domains, as

$$ {MNPPB}_i= mean\left\{{v}_i\right\}. $$

### Evaluation index of TAD-like domains

Given TAD-like domains *T = {T1, T2, T3, T4,…,Tn}* and contact matrix *F*_*i*, *j*_(*i*, *j* = 1, 2…*N*), we assess the structural property of domains by using the following two indices, according to the literature:

### Structure entropy (SE) [42]

$$ Num={\sum}_{1\le i\ne j\le N}{F}_{i,j};{T}_0=1;{T}_{n+1}=N+1, $$

$$ \mathrm{SE}\left(\mathrm{T},\mathrm{F}\right)=\raisebox{1ex}{${\sum}_{0\le i\le n}\frac{\sum_{T_i\le a<{T}_{i+1},b\ne a}{F}_{a,b}-{\sum}_{T_i\le a\ne b<{T}_{i+1}}{F}_{a,b}}{Num}\ast \mathit{\log}\left(\frac{\sum_{T_i\le a<{T}_{i+1},b\ne a}{F}_{a,b}}{Num}\right)$}\!\left/ \!\raisebox{-1ex}{$ logN$}\right. $$

$$ +\raisebox{1ex}{${\sum}_{0\le i\le n}{\sum}_{T_i\le j<{T}_{i+1}}\frac{\sum_{b\ne j}{F}_{j,b}}{Num}\ast \mathit{\log}\left(\frac{\sum_{b\ne j}{F}_{j,b}}{\sum_{T_i\le a<{T}_{i+1},b\ne a}{F}_{a,b}}\right)$}\!\left/ \!\raisebox{-1ex}{$ logN$}\right.. $$

### Modularity index (M) [41]

After removing the entries in the diagonal of the contact matrix, we split the chromosome into 6-Mb nonoverlapping windows. We further removed windows with fewer than 100 intra-window contacts, as the method was designed for TAD assessment with sufficient data [41]. For each window, we consider domain boundaries in/of the region *S = {S0 = 0, S1, S2, S3, S4,…,Sm = 150}* and the log transform contact matrix of the region *E*_*i*, *j*_(*i*, *j* = 1, 2…150). Then we calculated the modularity of this region as follows. The modularity of each 6-Mb region was then averaged into a modularity index.

$$ {M}_{x,y}=\raisebox{1ex}{$\left({E}_{x,y}-\frac{\sum_{a\ne x}{E}_{x,a}\times {\sum}_{a\ne y}{E}_{y,a}}{\sum \limits_{1\le i\ne j\le 150}{E}_{i,j}}\right)$}\!\left/ \!\raisebox{-1ex}{$\sum \limits_{1\le i\ne j\le 150}{E}_{i,j}$}\right. $$

$$ Modularity={\sum}_{0\le i<m}{\sum}_{S_i\le x\ne y<{S}_{i+1}}{M}_{x,y} $$

### Unsupervised classification of single cells

The classification based on TAD-like domain similarity in chromosome *k* between *n* cells is performed by PCA of the *C*_*i*, *j*, *k*_(*i*, *j* = 1, 2, …, *n*), which is the self-Spearman correlation coefficient matrix of the similarity matrix *M*_*i*, *j*, *k*_(*i*, *j* = 1, 2, …, *n*), calculated as follows:

$$ {M}_{i,j,k}= WS\left({Domain}_{cell\#i},{Domain}_{cell\#j}\right) $$

$$ {C}_{i,j,k}= Spearman\left({\overrightarrow{M}}_{i,\ast, k},{\overrightarrow{M}}_{j,\ast, k}\right) $$

The classification based on TAD-like domain similarity in all chromosomes between cells is performed by PCA of the matrix *T*_*i*, *j*_(*i*, *j* = 1, 2, …, *n*) and calculated as follows:

$$ {\overrightarrow{V}}_{\ast, k}= PC1\left({C}_{\ast, \ast, k}\right) $$

$$ {S}_{i,k}= symbol\left({V}_{i,k}\right)=\left\{\begin{array}{c}1\  if\ {V}_{i,k}>0\\ {}0\  if\ {V}_{i,k}=0\\ {}-1\  if\ {V}_{i,k}<0\end{array}\right. $$

$$ {T}_{i,j}=\left\{\begin{array}{c}\left({\sum}_{k=1,2,\dots }{S}_{i,k}\ast {S}_{j,k}\right)/\left({\sum}_{k=1,2,\dots}\left|{S}_{i,k}\ast {S}_{j,k}\right|\right), if\ {\sum}_{k=1,2,\dots}\left|{S}_{i,k}\ast {S}_{j,k}\right|>0\\ {}0, othe\mathrm{r} wise\end{array}\right.. $$

If data in chromosome *k* of cell *i* are not available, then *S*_*i*, *k*_will be set to 0, and the *T*s can only be calculated when both cells have sufficient data available.

After calculating PC1 of matrix *T*_*i*, *j*_(*i*, *j* = 1, 2, …, *n*), we get the classification index of each cell. Last, we access the classification by calculating the AUC of the ROC curve.

### The definition of matched, merged, split, and shifted TADs

As previously defined [65], the TAD boundary regions were defined as the flanking 100-kb region of the boundary bins, and the region between these TAD boundary regions was considered as being inside the TADs.

- Matched TADs: if both boundaries of a TAD in one condition aligned within TAD boundary regions in another condition.
- Merged TADs: if two or more TADs in one condition aligned inside of a TAD in another condition.
- Split TADs: if one boundary aligns to a boundary region of one TAD and the other boundary aligns inside of a different TAD.
- Shifted TADs: if the two boundaries of a TAD align into two different TADs.

### The definition of nested TAD boundary, unnested TAD boundary, and compartment domain boundary

Using Rao’s data at 40 kb resolution in chromosome one [18], we called TADs with deTOKI and obtained 253 TADs. Then the TAD boundaries were sorted by the number of contacts between the up- and downstream 400 kb regions, and 20 boundaries with the least number of cross-boundary contacts were removed. This was done because the removed boundaries are mostly located in the unmappable genome region. The top 20 TAD boundaries with highest cross-boundary contacts were defined as nested TAD boundaries, and the bottom 20 were defined as unnested TAD boundaries. The threshold of 20 does not substantially affect the results, as we showed in Additional file 2: Fig. S6b, in which the threshold of 40 was used. A compartment domain boundary was defined as the TAD boundaries that had different compartment scores between the flanking bins.

### Definition of 2i-specific TAD-like domain boundary and serum-specific TAD-like domain boundary

For 103 serum cells and 47 2i cells [55], we defined the bias of one bin as the difference in proportion between the serum cells and the 2i cells for which this bin represents a TAD-like domain boundary. We sorted all 40-kb bins in whole genome according to the bias value and defined the top and bottom 400 bins as serum- and 2i-specific TAD-like domain boundaries, respectively.

## Supplementary Information

**Additional file 1:** Supplemental Note.

**Additional file 2:** Supplementary figures and supplementary figure legends.

**Additional file 3:** Table S1. The statistics of predicted TAD-like domains using three methods with Tan’s data.

**Additional file 4:** Table S2. The statistics of predicted TAD-like domains using three methods with Flyamer’s data.

**Additional file 5:** Table S3. The statistics of predicted TAD-like domains using three methods with Li’s data.

**Additional file 6:** Table S4. CPU/GPU running times with single cell Hi-C of GM12878 cells in Tan’s data.

**Additional file 7:** Table S5. The description of used Hi-C and single-cell Hi-C data.

**Additional file 8:** Review history.

## Data Availability

The source code can be freely accessed at Github [66], and at the repository Zenodo [67], under a GPLv3 license. The ensemble Hi-C data is available from GEO under accession numbers GSE35156 [14] and GSE63525 [18]. The single-cell Hi-C data is available from GEO under accession number GSE117876 [54], GSE80006 [50], and GSE119171 [55]. All simulated and experimental data used in this study are summarized in Additional file 7: Table S5.
