# Supplementary material for: DeTOKI identifies and characterizes the dynamics of chromatin TAD-like domains in a single cell
Source: Genome Biol. 2021 Jul 27;22:217. doi: 10.1186/s13059-021-02435-7 (PMC8314462; doi:10.1186/s13059-021-02435-7)
Supplement: Supplementary file 1 — Additional file 1: Supplemental Note. [file 13059_2021_2435_MOESM1_ESM.docx]

Supplementary Text

**DeTOKI identifies and characterizes the dynamics of chromatin TAD-like domains in a single cell**

Xiao Li ^1,2,3^, Guangjie Zeng^4^, Angsheng Li^4^ and Zhihua Zhang^1,2,3^*

^1^ CAS Key Laboratory of Genome Sciences and Information, Beijing Institute of Genomics, Chinese Academy of Sciences, and China National Center for Bioinformation, Beijing 100101, China

^2^ School of Life Science, University of Chinese Academy of Sciences, Beijing, P.R. China

^3^ School of Artificial Intelligence，University of Chinese Academy of Sciences, Beijing, P.R. China

^4^ State Key Laboratory of Software Development Environment, School of Computer Science, Beihang University, 100083 Beijing, P.R. China.

* Correspondence should be addressed to Zhihua Zhang at [zhangzhihua@big.ac.cn](mailto:zhangzhihua@big.ac.cn).

**The performance of deTOKI in bulk Hi-C and simulated Hi-C**

We validate the efficiency of deTOKI in Dixon’s Hi-C data. It shows that TAD boundaries locate on the boundaries of block pattern in contact matrix (Supplementary Fig. 1a). Aggregate analysis of deTOKI-predicted TADs also reveals the enrichment of functional signaling on boundaries, including CTCF, H3K4me3, and H3K36me3. (Methods; Supplementary Fig. 2a-c). To cross- validate the accuracy of deTOKI’s boundary, we compare the similarity between it and the results of other TAD algorithms, namely Insulation Score (IS) and deDoc (Methods). deTOKI has high similarity with IS, a widespread method used in recent years (Supplementary Fig.2d). The deTOKI also performs well in simulated Hi-C data based on CTCF motif. It is well known that CTCF motif often shows convergent orientation at chromatin interactions, as well as bias of orientation upstream or downstream of TAD boundary. Therefore, we define “motif insulation strength” to simulate a Hi-C contact matrix and perform deTOKI (Methods). As expected, the detected domains based on CTCF motif are very similar to TADs detected from Hi-C data (Supplementary Fig.2e).

To simulate ensemble Hi-C data based on CTCF motif, we define motif insulation strength between ${bin}_{i}$ and ${bin}_{j}$, as follows:

${Insulation}_{ij}={max}_{i\leq k\leq j}\left( \sum_{i\leq x<k} {BM}_{x}+\sum_{k\leq x<j} {FM}_{x} \right)$,

where${BM}_{x}$, ${FM}_{x}$ denote the backward and forward motif number in${bin}_{x}$, respectively. Then we perform Poisson regression on existing Hi-C data as follows to simulate the Hi-C data:

${Contact number}_{ij}\sim1+abs\left( j-i \right)+{Insulation}_{ij}$.

**On the reliability of deTOKI under sub-single-cell Hi-C level data**

Taking the data from cell #11 of GM12878 of Tan’s data as the example, we searched for the minimal reads count needed for reliable TAD-like domain prediction with deTOKI by downsampling. Three downsample rates, i.e., 1/10, 1/30 and 1/100, were used in this analysis, representing about 0.11M, 0.04M and 0.01M reads per cell, respectively. To assess the reliability of the predictions, we compared the similarity between the domain predicted from downsampled data and full data of cell #11 versus the similarities between the downsampled data and other cells from Tan. We defined a domain prediction downsample rate as reliable If the similarity of predicted domain to cell #11 remains significantly larger than its similarity to other cells. We found deTOKI remains reliable under sample rate 1/10, but loses its reliability with fewer reads (Supplementary Fig.9). Thus, the recommended minimal data for TAD-like domain prediction for deTOKI is 0.1M per cell at 40kb resolution.

**The comparison between deTOKI and Higashi**

A recently published single-cell Hi-C analysis toolkit named Higashi, predicts TAD-like domains on the imputed single-cell Hi-C data with hypergraph representation learning. To assess the improvement that data imputation may introduce to TAD-like domain predictions, we compared the performance of deTOKI, deTOKI with data imputation by scHiCluster, denoted as deTOKI+scHiCluster, and Higashi.

First, we compared the three tools with the downsampled 1/800 data of Dixon et al. and found comparable performance among the three. The largest absolute log2 fold changes (|log2FC|) in predicted TAD-like domain numbers between the downsampled datasets were all less than 0.3, and deTOKI+scHiCluster had the lowest |log2FC| on average (Supplementary Fig.10a) Taking the TADs identified with the full data as the gold standard, we compared the adjusted mutual information (AMI) and weighted similarity (WS) among the tools. All three tools had AMI and WS values rather close to each other with AMIs all around 0.8, on average, and WS about 0.71 (deTOKI+scHiCluster and Higashi were slightly better with average WS=0.74, Supplementary Fig.10b).

Second, we compared the three tools with simulated single-cell Hi-C data with setting identical to that in the main text between the genome region of chr18:50-55Mb and 10-15Mb (Supplementary Fig.10c). For all four indices assessed (AMI, WS, VI and BP), we found that the three tools performed in a similar manner. In the region chr18: 50-55Mb, deTOKI and deTOKI+scHiCluster showed better performance on all four indices than Higashi. In the region chr18: 10-15Mb, Higashi and deTOKI+scHiCluster had better performance than deTOKI alone in about half the settings. Last, for the classification task, deTOKI+scHiCluster had the lowest misclassification number compared to Higashi or deTOKI alone (Supplementary Fig.10d).

Taken together, deTOKI and Higashi have similar performance and deTOKI can be further improved by data imputation, e.g., scHiCluster. Considering the substantial CPU time required by Higashi (about 100-fold more CPU time than that required by either deTOKI or scHiCluster; Supplementary Table4), deTOKI is more efficient on TAD-like domain identification with single-cell Hi-C data.

**Supplementary Figure legends**

**Supplementary Fig.1** **A** The predicted TAD-like domains under different “k” (NMF times) in four examples, including contact matrices of ensemble Hi-C data (in Dixon et al.) and single-cell Hi-C data (in Tan et al.). Predicted TAD-like domains are shown in sawtooth. **B** The predicted TAD-like domains under different resolution in two examples, including contact matrices of ensemble Hi-C data (in Dixon et al.) and single-cell Hi-C data (in Tan et al.). Predicted domains are shown in sawtooth. **C** The left and right scatter plots represent running time of deTOKI using 1 core or 16 cores, respectively. Each point represents an intra-chromosome Hi-C contact matrix from oocytes ensemble Hi-C data (in Flyamer et al.).

**Supplementary Fig.2** deTOKI can accurately detect TADs in ensemble Hi-C data. **A-C** The plots represent the expectancy of ChIP-seq peaks with CTCF, H3K4me3, and H3k36me3 on the predicted ensemble TAD boundaries (chr1-22), respectively. The *y*-axis represents the mean number of peaks per bin with the same distance to the predicted TAD boundaries. The shadow represents 95% confidence interval as calculated by bootstrap. The p value is resulted from permutation test on enrichment of ChIP-seq peaks on TAD boundaries. **D** The radar plot shows the similarities between the TADs predicted by the different algorithms. Each spoke represents a comparison of AMIs between a reference algorithm (indicated as a colored square) and each of the other algorithms. Abbreviations: IS (Insulation Score), DD (deDoc), MT (MrTADFinder), HM (HOMER), AT (Armatus), HS (HiCseg), TK (deTOKI). **E** An example of simulated data based on CTCF motifs. Heatmap of the Hi-C contact matrix from bulk Hi-C data, and simulated data are the upper part and lower part, respectively. Predicted TADs and CTCF domains are shown in blue sawtooth and green sawtooth, respectively.

**Supplementary Fig.3** Comparison of TAD predictors in down-sampled (**A-D**) and simulated (**E-H**) single-cell Hi-C data. **A** The differences of TAD-like domains, as inferred by BP and VI, between raw data and down-sampled data in different chromosomes. **B** The (log2) change on number of predicted domains in different chromosomes on 20kb bin-size and 80kb bin-size. **C** The similarity and differences of domains, as inferred by each index, between raw data and down-sampled data in different chromosomes on 20kb bin-size and 80kb bin-size. **D** The genome-wide distribution of ChIP-seq peaks of CTCF, H3K4me3 and H3K36me3 flanking the predicted domain boundaries**,** respectively. The shadow represents 95% confidence interval as calculated by bootstrap. The *y*-axis represents the mean number of peaks per bin with the same distance to the predicted domain boundaries (MNPPB). The enrichment p values are calculated by permutation test (n=10000). **E** From left to right, the normalized Hi-C contact matrix of chr18:10-15Mb for GM12878 ensemble Hi-C from Rao’s data^18^, an ensemble of 100 modeled 3D structures of this region, and the 3D structure modeled from the simulated ensemble Hi-C from model #100. Each dot in the right panel represents a particle 10kb long, and the dots with same color belong to the same predicted ensemble TAD. **F** The differences of predicted single-cell domains between different thresholds and predictors on chr18:50-55Mb. **G** The cumulative distribution function of distance between bin pairs in the representative example (model#1). **H** The similarities and differences of predicted single-cell domains between different thresholds and predictors on chr18:10-15Mb. *: P<0.05, **: P<0.001, NS: not significant, two-sided Mann-Whitney U test.

**Supplementary Fig.4** deTOKI performs well in real single-cell Hi-C data. **A** Radar plots on the left and right panel show Modularity Index and Structure Entropy of predicted TAD-like domains by each software program on chr1 of 30 oocytes and 10 zygotes-mat (in Flyamer et al.) and on chr1 of 150 mESCs (in Li et al.), respectively. **B-C** The probability mass function of length of predicted domains by deTOKI and IS in single-cell Hi-C data (PBMC cell#14 chr1) and its down-sampled half data. **D** An example of single-cell data (in Tan et al.) and its down-sampled data at half level. Heatmap of the Hi-C contact matrix from single-cell Hi-C data; down-sampled data are the upper and lower panels, respectively. Predicted domains in each data are shown in sawtooth. **E** The similarities between predicted domains in several single-cell Hi-C data and their down-sampled data at half level by predictors. **F** The probability mass function of domain length predicted by deTOKI and IS in chr1-22 (in Tan et al.). **G** The mean contact coverage (in Tan et al.) on mini domains predicted by IS and other domains in chr1-22. *: P<0.05, **: P<0.001, NS: not significant, two-sided Mann-Whitney U test.

**Supplementary Fig.5** TAD-like domain structure is highly dynamic at the single-cell level. **A** The cell-to-cell and cell-to-ensemble similarity of deTOKI-predicted domains. The single-cell data was from 30 oocytes (in Flyamer et al.), compared to the ensemble in mESC and oocyte. **B** The cell-to-cell and cell-to-ensemble similarity of deTOKI-predicted domains. The single-cell data was from 150 mESCs (in Li et al.), compared to the ensemble in mESC and oocyte. **C** The diagram of four types of TAD changes in a single cell. **D** Distribution of different types of ensemble TADs in chr1 (in Tan et al.). **E** Example of predicted single-cell domains and ensemble TADs. The type of ensemble TAD is marked in color. *: P<0.05, **: P<0.001, NS: not significant, two-sided Wilcoxon rank-sum test.

**Supplementary Fig.6** The ensemble TAD boundaries were not purely randomly distributed in single cells. **A** Number of cells in which the ensemble TAD boundary is also a TAD-like domain boundary. The statistic is shown for four types of ensemble TAD boundaries. The p value was calculated by two-sided Wilcoxon rank-sum test. **B**. Number of cells in which the ensemble TAD boundary is also a TAD-like domain boundary. The statistic is shown for nested and unnested ensemble TAD boundaries under threshold 40. The p value was calculated by two-sided Wilcoxon rank-sum test. **C.** The distribution of number of cross-boundary contacts versus the number of cells that appeared in the single cells**.** **D, E and F** GO analysis of genes on the over- and under-represented boundaries and other ensemble TAD boundaries, respectively.

**Supplementary Fig.7** The scSBs may not fully result from stochastic fluctuation. **A** The distribution of histone marks flanking the deTOKI-, IS- or deDoc-predicted single-cell boundaries is shown, respectively**. B** The distribution of histone marks flanking the deTOKI-, IS- or deDoc- predicted ensemble boundaries are shown, respectively**. C** The distribution of histone marks flanking the deTOKI-predicted scSB-m, scSB-1 and -2 domain boundaries are shown, respectively**.** The *y*-axis of panel **(A-C)** represents the mean number of peaks per bin with the same distance to the predicted domain boundaries normalized by average in whole genome (MNPPB). The shadow represents 95% confidence interval, as calculated by bootstrap. **D** The enrichment p values and consensus p values (Methods) of each histone mark on single cell domain boundaries and ensemble TAD boundaries predicted by deTOKI. **E** enrichment p values and consensus p values (Methods) of each histone mark on scSBs-m and scSBs-1,2. **F** The average number of ChIP-seq peaks in scSBs. **G** The distance to the nearest ensemble boundaries of scSBs in each class. **H** A logistic regression model to classify scSB-1,2 and scSB-m based on 12 Chip-seq peaks. Factors with a positive coefficient have a direct effect on scSB-m. Only the significant factors are displayed. *: P<0.05, **: P<0.001, NS: not significant, two-sided Wilcoxon rank-sum test.

**Supplementary Fig.8** TAD-like domain structure carries the information for the cell identity. **A-B** GO analysis of genes on the serum-specific single-cell domain boundaries and genes on the 2i-specific single-cell TAD boundaries, respectively, in Li’s dataset. **C** The number of ChIP-seq peaks on two types of single-cell domain boundaries. **D** The PCC of DNA methylation rate in bin pairs cross ensemble TAD boundaries which have a weak insulation score, and bin pairs cross ensemble TAD boundaries which have strong insulation score. **E** The classification of single cells based on predicted domain boundaries in Flyamer’s datasets. The x- and y-axis represent the PC1 calculated by deTOKI and IS, respectively. The embedded plots show the AUC of classification by each program. *: P<0.05, **: P<0.001, Fisher’s z-test.

**Supplementary Fig.9** The similarities of deTOKI-predicted TAD-like domains, as inferred by AMI (A and C) and WS (B and D), between downsampled and raw data from cell #11 or between downsampled cell #11 data and data of other cells in chr1 and chr10. *: P<0.05, **: P<0.001, NS: not significant, two-sided Wilcoxon rank-sum test.

**Supplementary Fig.10** Comparison of deTOKI and Higashi on downsampled and simulated single-cell Hi-C based on data from IMR90. Panels (A) and (B) show the average results of 20 independent downsamplings in each chromosome. **(A)** The (log2) change in the number of predicted TAD-like domains. **(B)** The similarity of TAD-like domains, as inferred by AMI and WS, between the raw data and the downsampled data. **(C)** Similarities (AMI and WS) and differences (VI and BP) of predicted single-cell TAD-like domains between different thresholds and predictors. **(D)** Number of misclassifications, using predicted TAD-like domains.

**Supplementary Table 1** The statistics of predicted TAD-like domains using three methods with Tan’s data.

The statistics includes number and length of predicted domains using IS, deDoc and deTOKI. Each row represents a cell on Tan’s data. GM12878#8 was eliminated because it doesn’t have cleaned data on GEO.

**Supplementary Table 2** The statistics of predicted TAD-like domains using three methods with Flyamer’s data.

The statistics includes number and length of predicted domains using IS, deDoc and deTOKI. Each row represents a cell on Flyamer’s data. The names consist with sample names on GEO.

**Supplementary Table 3** The statistics of predicted TAD-like domains using three methods with Li’s data.

The statistics includes number and length of predicted domains using IS, deDoc and deTOKI. Each row represents a cell on Li’s data.

**Supplementary Table 4** CPU/GPU running times with single cell Hi-C of GM12878 cells in Tan’s data.

**Supplementary Table 5** The description of used Hi-C and single-cell Hi-C data.

The description includes cell type, resolution of contact matrix, contacts number, sparsity, dynamic range of contacts and availability of data. The calculation method of sparsity and dynamic range is explained at the bottom of the table.
