# Supplementary material for: DeTOKI identifies and characterizes the dynamics of chromatin TAD-like domains in a single cell
Source: Genome Biol. 2021 Jul 27;22:217. doi: 10.1186/s13059-021-02435-7 (PMC8314462; doi:10.1186/s13059-021-02435-7)
Supplement: Supplementary file 2 — Additional file 2: Supplementary figures and supplementary figure legends. [file 13059_2021_2435_MOESM2_ESM.pdf]

Supplementary Figure legends

Supplementary Fig.1 A The predicted TAD-like domains under different "k" (NMF times) in four examples, including contact matrices of ensemble Hi-C data (in Dixon et al.) and single-cell Hi-C data (in Tan et al.). Predicted TAD-like domains are shown in sawtooth. **B** The predicted TAD-like domains under different resolution in two examples, including contact matrices of ensemble Hi-C data (in Dixon et al.) and single-cell Hi-C data (in Tan et al.). Predicted domains are shown in sawtooth. **C** The left and right scatter plots represent running time of deTOKI using 1 core or 16 cores, respectively. Each point represents an intra-chromosome Hi-C contact matrix from oocytes ensemble Hi-C data (in Flyamer et al.).

Supplementary Fig.2 deTOKI can accurately detect TADs in ensemble Hi-C data. **A-C** The plots represent the expectancy of ChIP-seq peaks with CTCF, H3K4me3, and H3k36me3 on the predicted ensemble TAD boundaries (chr1-22), respectively. The *y*-axis represents the mean number of peaks per bin with the same distance to the predicted TAD boundaries. The shadow represents 95% confidence interval as calculated by bootstrap. The p value is resulted from permutation test on enrichment of ChIP-seq peaks on TAD boundaries. **D** The radar plot shows the similarities between the TADs predicted by the different algorithms. Each spoke represents a comparison of AMIs between a reference algorithm (indicated as a colored square) and each of the other algorithms. Abbreviations: IS (Insulation Score), DD (deDoc), MT (MrTADFinder), HM (HOMER), AT (Armatus), HS (HiCseg), TK (deTOKI). **E** An example of simulated data based on CTCF motifs. Heatmap of the Hi-C contact matrix from bulk Hi-C data, and simulated data are the upper part and lower part, respectively. Predicted TADs and CTCF domains are shown in blue sawtooth and green sawtooth, respectively.

Supplementary Fig.3 Comparison of TAD predictors in down-sampled (**A-D**) and simulated (**E-H**) single-cell Hi-C data. **A** The differences of TAD-like domains, as inferred by BP and VI, between raw data and down-sampled data in different chromosomes. **B** The (log2) change on number of predicted domains in different chromosomes on 20kb bin-size and 80kb bin-size. **C** The similarity and differences of domains, as inferred by each index, between raw data and down-sampled data in different chromosomes on 20kb bin-size and 80kb bin-size. **D** The genome-wide distribution of ChIP-seq peaks of CTCF, H3K4me3 and H3K36me3 flanking the predicted domain boundaries, respectively. The shadow represents 95% confidence interval as calculated by bootstrap. The *y*-axis represents the mean number of peaks per bin with the same distance to the predicted domain boundaries (MNPPB). The enrichment p values are calculated by permutation test (n=10000). **E** From left to right, the normalized Hi-C contact matrix of chr18:10-15Mb for GM12878 ensemble Hi-C from Rao's data[18], an ensemble of 100 modeled 3D structures of this region,

and the 3D structure modeled from the simulated ensemble Hi-C from model #100. Each dot in the right panel represents a particle 10kb long, and the dots with same color belong to the same predicted ensemble TAD. **F** The differences of predicted single-cell domains between different thresholds and predictors on chr18:50-55Mb. **G** The cumulative distribution function of distance between bin pairs in the representative example (model#1). **H** The similarities and differences of predicted single-cell domains between different thresholds and predictors on chr18:10-15Mb. *: P<0.05, **: P<0.001, NS: not significant, two-sided Mann-Whitney U test.

Supplementary Fig.4 deTOKI performs well in real single-cell Hi-C data. **A** Radar plots on the left and right panel show Modularity Index and Structure Entropy of predicted TAD-like domains by each software program on chr1 of 30 oocytes and 10 zygotes-mat (in Flyamer et al.) and on chr1 of 150 mESCs (in Li et al.), respectively. **B-C** The probability mass function of length of predicted domains by deTOKI and IS in single-cell Hi-C data (PBMC cell#14 chr1) and its down-sampled half data. **D** An example of single-cell data (in Tan et al.) and its down-sampled data at half level. Heatmap of the Hi-C contact matrix from single-cell Hi-C data; down-sampled data are the upper and lower panels, respectively. Predicted domains in each data are shown in sawtooth. **E** The similarities between predicted domains in several single-cell Hi-C data and their down-sampled data at half level by predictors. **F** The probability mass function of domain length predicted by deTOKI and IS in chr1-22 (in Tan et al.). **G** The mean contact coverage (in Tan et al.) on mini domains predicted by IS and other domains in chr1-22. *: P<0.05, **: P<0.001, NS: not significant, two-sided Mann-Whitney U test.

Supplementary Fig.5 TAD-like domain structure is highly dynamic at the single-cell level. **A** The cell-to-cell and cell-to-ensemble similarity of deTOKI-predicted domains. The single-cell data was from 30 oocytes (in Flyamer et al.), compared to the ensemble in mESC and oocyte. **B** The cell-to-cell and cell-to-ensemble similarity of deTOKI-predicted domains. The single-cell data was from 150 mESCs (in Li et al.), compared to the ensemble in mESC and oocyte. **C** The diagram of four types of TAD changes in a single cell. **D** Distribution of different types of ensemble TADs in chr1 (in Tan et al.). **E** Example of predicted single-cell domains and ensemble TADs. The type of ensemble TAD is marked in color. *: P<0.05, **: P<0.001, NS: not significant, two-sided Wilcoxon rank-sum test.

Supplementary Fig.6 The ensemble TAD boundaries were not purely randomly distributed in single cells. **A** Number of cells in which the ensemble TAD boundary is also a TAD-like domain boundary. The statistic is shown for four types of ensemble TAD boundaries. The p value was calculated by two-

sided Wilcoxon rank-sum test. **B**. Number of cells in which the ensemble TAD boundary is also a TAD-like domain boundary. The statistic is shown for nested and unnested ensemble TAD boundaries under threshold 40. The p value was calculated by two-sided Wilcoxon rank-sum test. **C.** The distribution of number of cross-boundary contacts versus the number of cells that appeared in the single cells**. D, E and F** GO analysis of genes on the over- and under-represented boundaries and other ensemble TAD boundaries, respectively.

Supplementary Fig.7 The scSBs may not fully result from stochastic fluctuation. **A** The distribution of histone marks flanking the deTOKI-, IS- or deDoc-predicted single-cell boundaries is shown, respectively**. B** The distribution of histone marks flanking the deTOKI-, IS- or deDoc- predicted ensemble boundaries are shown, respectively**. C** The distribution of histone marks flanking the deTOKI-predicted scSB-m, scSB-1 and -2 domain boundaries are shown, respectively**.** The *y*-axis of panel **(A-C)** represents the mean number of peaks per bin with the same distance to the predicted domain boundaries normalized by average in whole genome (MNPPB). The shadow represents 95% confidence interval, as calculated by bootstrap. **D** The enrichment p values and consensus p values (Methods) of each histone mark on single cell domain boundaries and ensemble TAD boundaries predicted by deTOKI. **E** enrichment p values and consensus p values (Methods) of each histone mark on scSBs-m and scSBs-1,2. **F** The average number of ChIP-seq peaks in scSBs. **G** The distance to the nearest ensemble boundaries of scSBs in each class. **H** A logistic regression model to classify scSB-1,2 and scSB-m based on 12 Chip-seq peaks. Factors with a positive coefficient have a direct effect on scSB-m. Only the significant factors are displayed. *: P<0.05, **: P<0.001, NS: not significant, two-sided Wilcoxon rank-sum test.

Supplementary Fig.8 TAD-like domain structure carries the information for the cell identity. **A-B** GO analysis of genes on the serum-specific single-cell domain boundaries and genes on the 2i-specific single-cell TAD boundaries, respectively, in Li's dataset. **C** The number of ChIP-seq peaks on two types of single-cell domain boundaries. **D** The PCC of DNA methylation rate in bin pairs cross ensemble TAD boundaries which have a weak insulation score, and bin pairs cross ensemble TAD boundaries which have strong insulation score. **E** The classification of single cells based on predicted domain boundaries in Flyamer's datasets. The x- and y-axis represent the PC1 calculated by deTOKI and IS, respectively. The embedded plots show the AUC of classification by each program. *: P<0.05, **: P<0.001, Fisher's z-test.

Supplementary Fig.9 The similarities of deTOKI-predicted TAD-like domains, as inferred by AMI (A and C) and WS (B and D), between downsampled and raw data from cell #11 or between downsampled cell #11 data and data of other cells in chr1 and chr10. *: P<0.05, **: P<0.001, NS: not significant, two-sided

Wilcoxon rank-sum test.

Supplementary Fig.10 Comparison of deTOKI and Higashi on downsampled and simulated single-cell Hi-C based on data from IMR90. Panels (A) and (B) show the average results of 20 independent downsamplings in each chromosome. **(A)** The (log2) change in the number of predicted TAD-like domains. **(B)** The similarity of TAD-like domains, as inferred by AMI and WS, between the raw data and the downsampled data. **(C)** Similarities (AMI and WS) and differences (VI and BP) of predicted single-cell TAD-like domains between different thresholds and predictors. **(D)** Number of misclassifications, using predicted TAD-like domains.

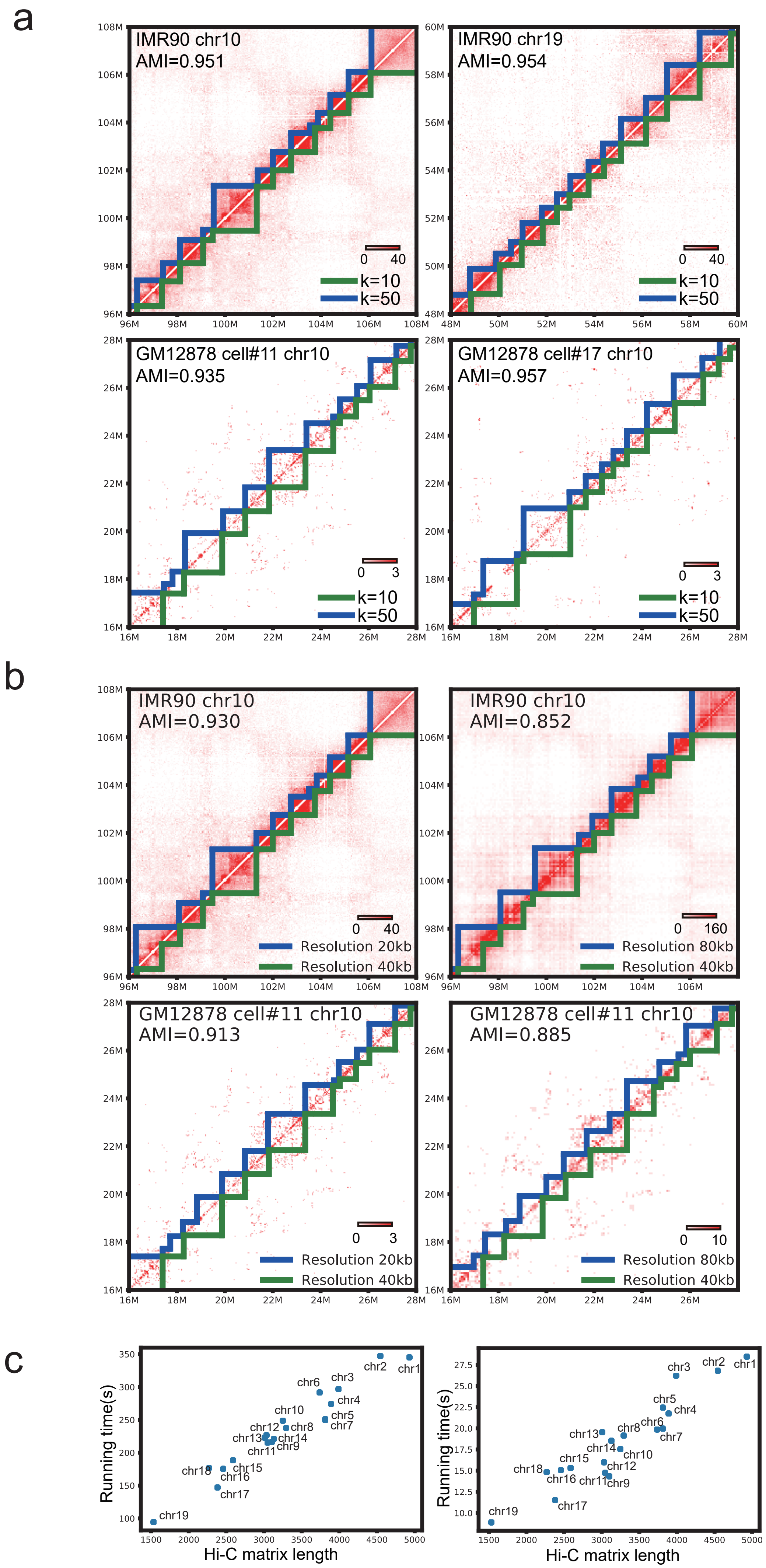
a
IMR90 chr10
AMI=0.951
IMR90 chr19
AMI=0.954
GM12878 cell#11 chr10
AMI=0.935
GM12878 cell#17 chr10
AMI=0.957
k=10
k=50
b
IMR90 chr10
AMI=0.930
IMR90 chr10
AMI=0.852
GM12878 cell#11 chr10
AMI=0.913
GM12878 cell#11 chr10
AMI=0.885
Resolution 20kb
Resolution 40kb
Resolution 80kb
c
Running time(s)
Hi-C matrix length
chr1
chr2
chr3
chr4
chr5
chr6
chr7
chr8
chr9
chr10
chr11
chr12
chr13
chr14
chr15
chr16
chr17
chr18
chr19

Figure S1

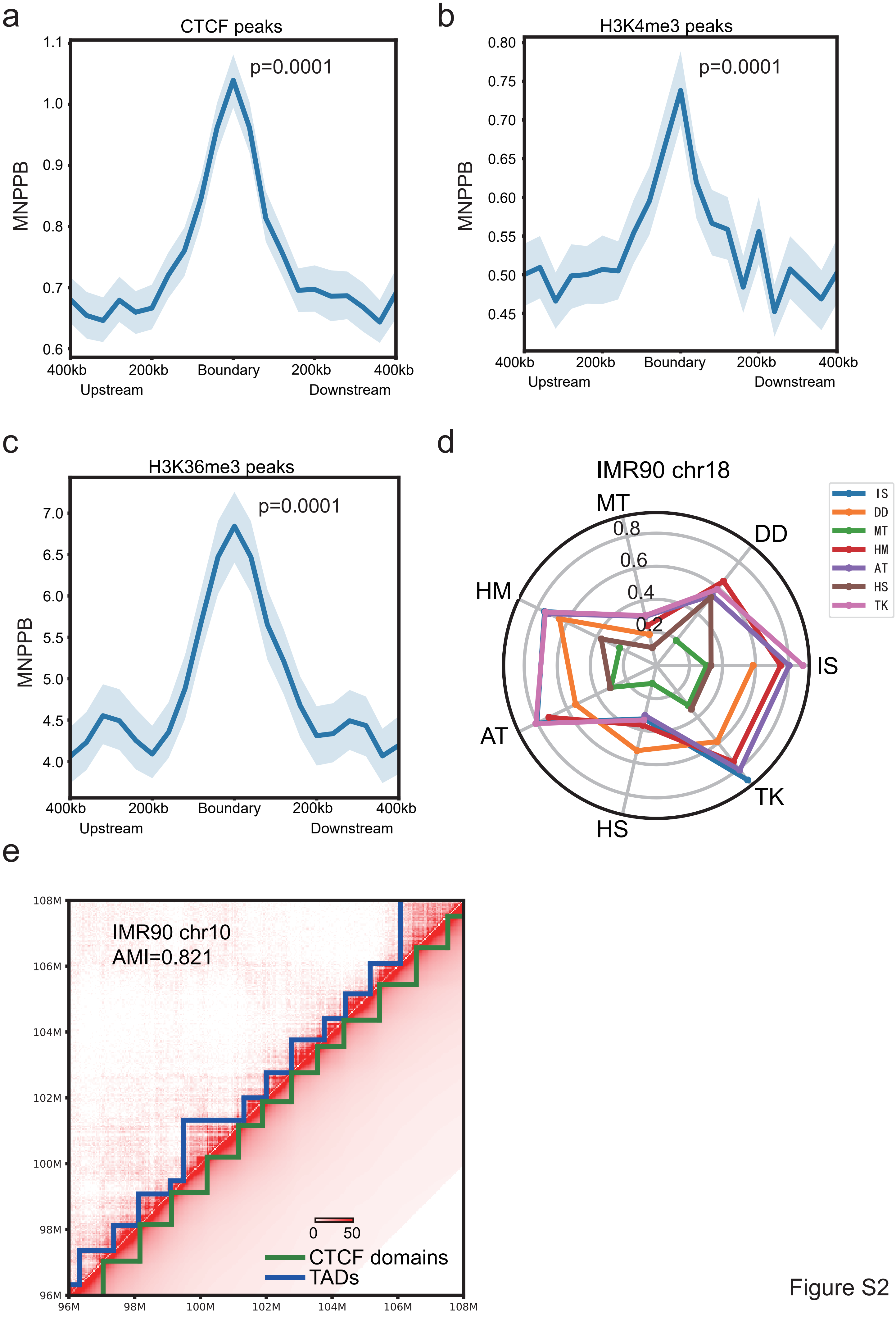
a
CTCF peaks
p=0.0001
MNPPB
1.1
1.0
0.9
0.8
0.7
0.6
400kb
200kb
Boundary
200kb
400kb
Upstream
Downstream
b
H3K4me3 peaks
p=0.0001
MNPPB
0.80
0.75
0.70
0.65
0.60
0.55
0.50
0.45
400kb
200kb
Boundary
200kb
400kb
Upstream
Downstream
c
H3K36me3 peaks
p=0.0001
MNPPB
7.0
6.5
6.0
5.5
5.0
4.5
4.0
400kb
200kb
Boundary
200kb
400kb
Upstream
Downstream
d
IMR90 chr18
MT
DD
IS
TK
HS
AT
HM
0.8
0.6
0.4
0.2
IS
DD
MT
HM
AT
HS
TK
e
IMR90 chr10
AMI=0.821
0
50
CTCF domains
TADs
108M
106M
104M
102M
100M
98M
96M
96M
98M
100M
102M
104M
106M
108M

Figure S2

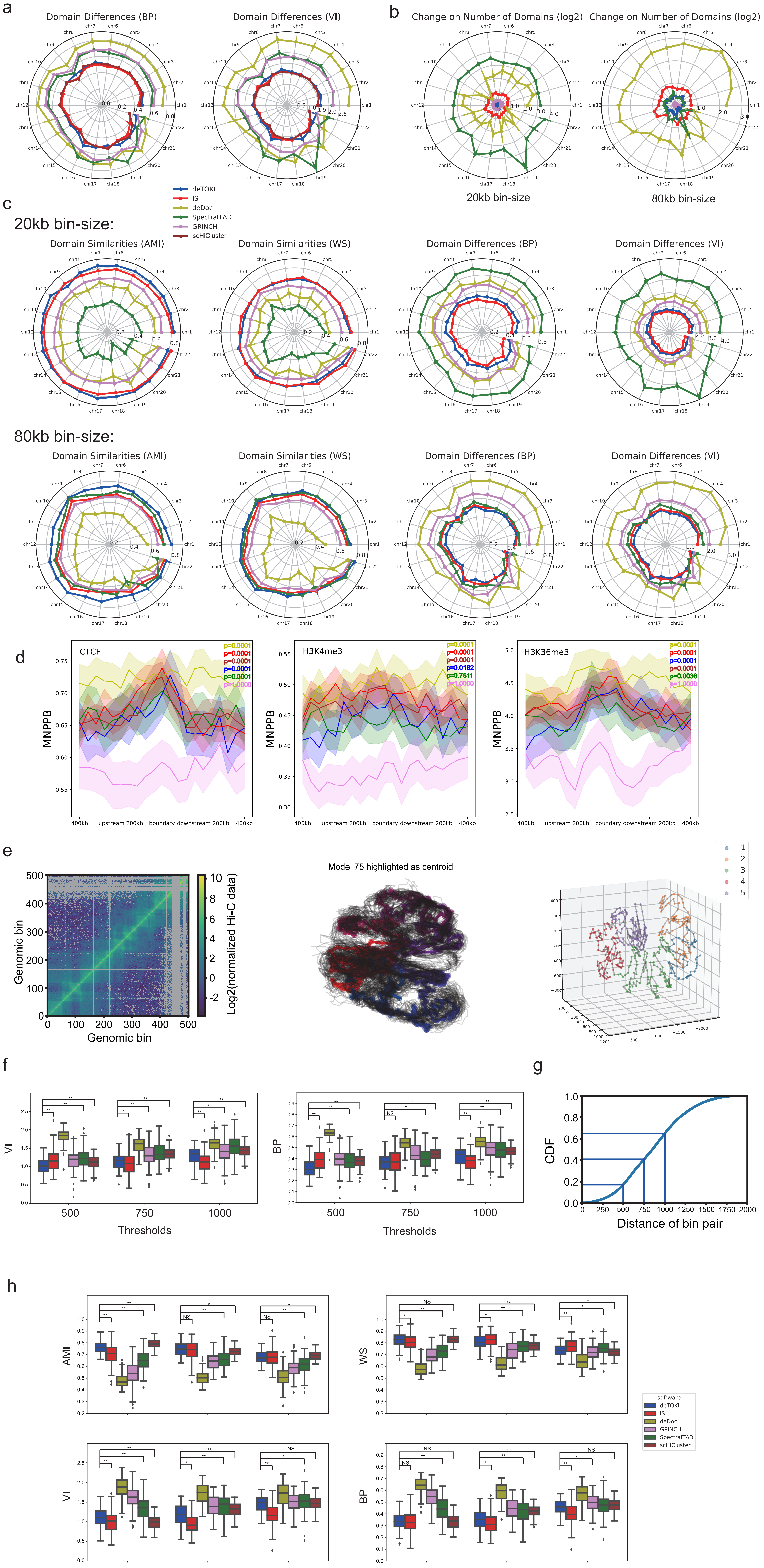
a
Domain Differences (BP)
Domain Differences (VI)
b
Change on Number of Domains (log2)
Change on Number of Domains (log2)
20kb bin-size
80kb bin-size
c
deTOKI
IS
deDoc
SpectralTAD
GRiNCH
scHiCluster
20kb bin-size:
Domain Similarities (AMI)
Domain Similarities (WS)
Domain Differences (BP)
Domain Differences (VI)
80kb bin-size:
Domain Similarities (AMI)
Domain Similarities (WS)
Domain Differences (BP)
Domain Differences (VI)
d
CTCF
H3K4me3
H3K36me3
MNPPB
400kb
upstream 200kb
boundary
downstream 200kb
400kb
e
Genomic bin
Log2(normalized Hi-C data)
Model 75 highlighted as centroid
f
VI
BP
Thresholds
g
CDF
Distance of bin pair
h
AMI
WS
VI
BP
software
deTOKI
IS
deDoc
GRiNCH
SpectralTAD
scHiCluster

Figure S3

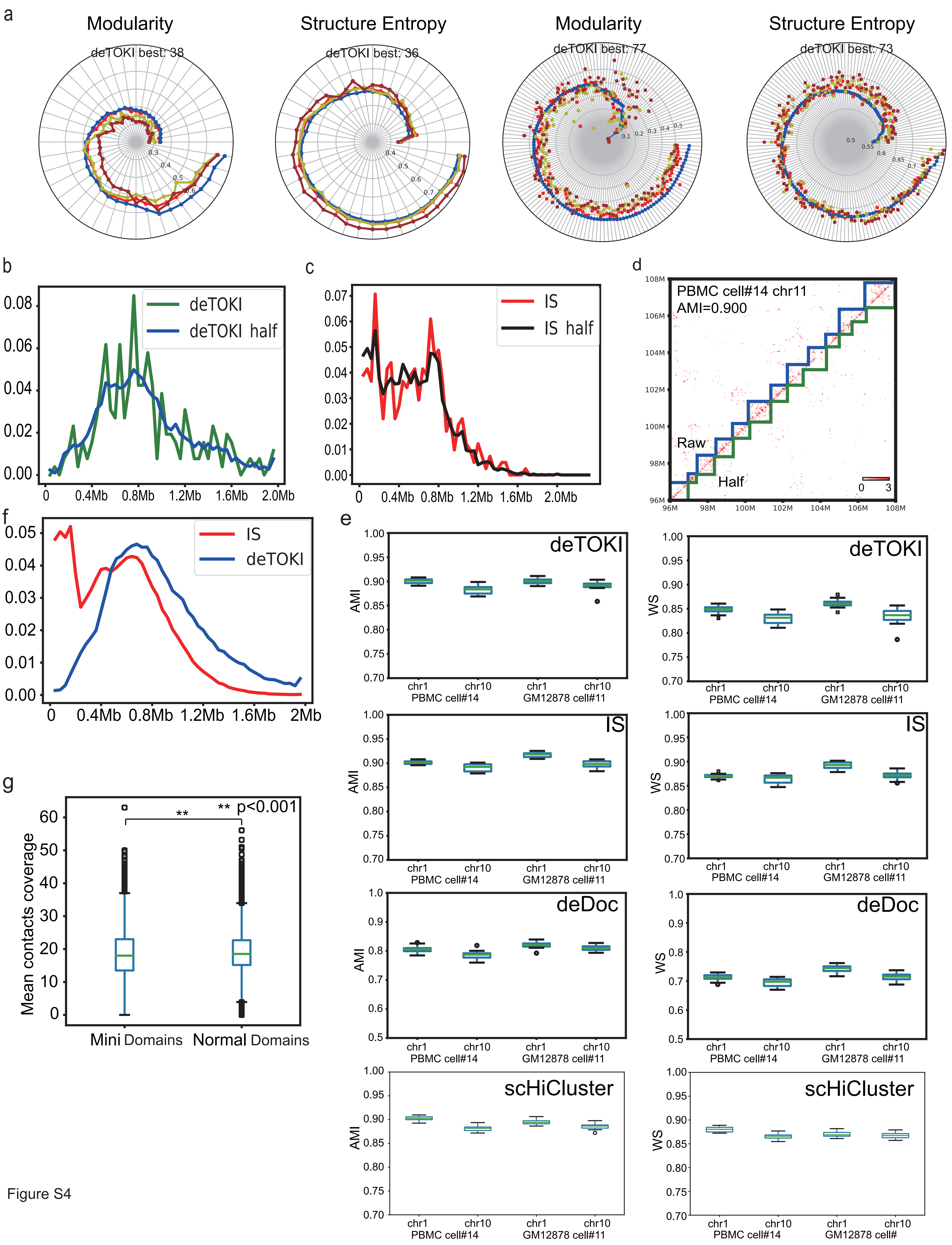
a
Modularity
deTOKI best: 38
Structure Entropy
deTOKI best: 36
Modularity
deTOKI best: 77
Structure Entropy
deTOKI best: 73
b
deTOKI
deTOKI half
0 0.4Mb 0.8Mb 1.2Mb 1.6Mb 2.0Mb
c
IS
IS half
0 0.4Mb 0.8Mb 1.2Mb 1.6Mb 2.0Mb
d
PBMC cell#14 chr11
AMI=0.900
Raw
Half
96M 98M 100M 102M 104M 106M 108M
f
IS
deTOKI
0 0.4Mb 0.8Mb 1.2Mb 1.6Mb 2Mb
e
deTOKI
IS
deDoc
scHiCluster
AMI
WS
chr1 chr10 chr1 chr10
PBMC cell#14 GM12878 cell#11
PBMC cell#14 GM12878 cell#
g
** p<0.001
**
Mean contacts coverage
Mini Domains
Normal Domains

Figure S4

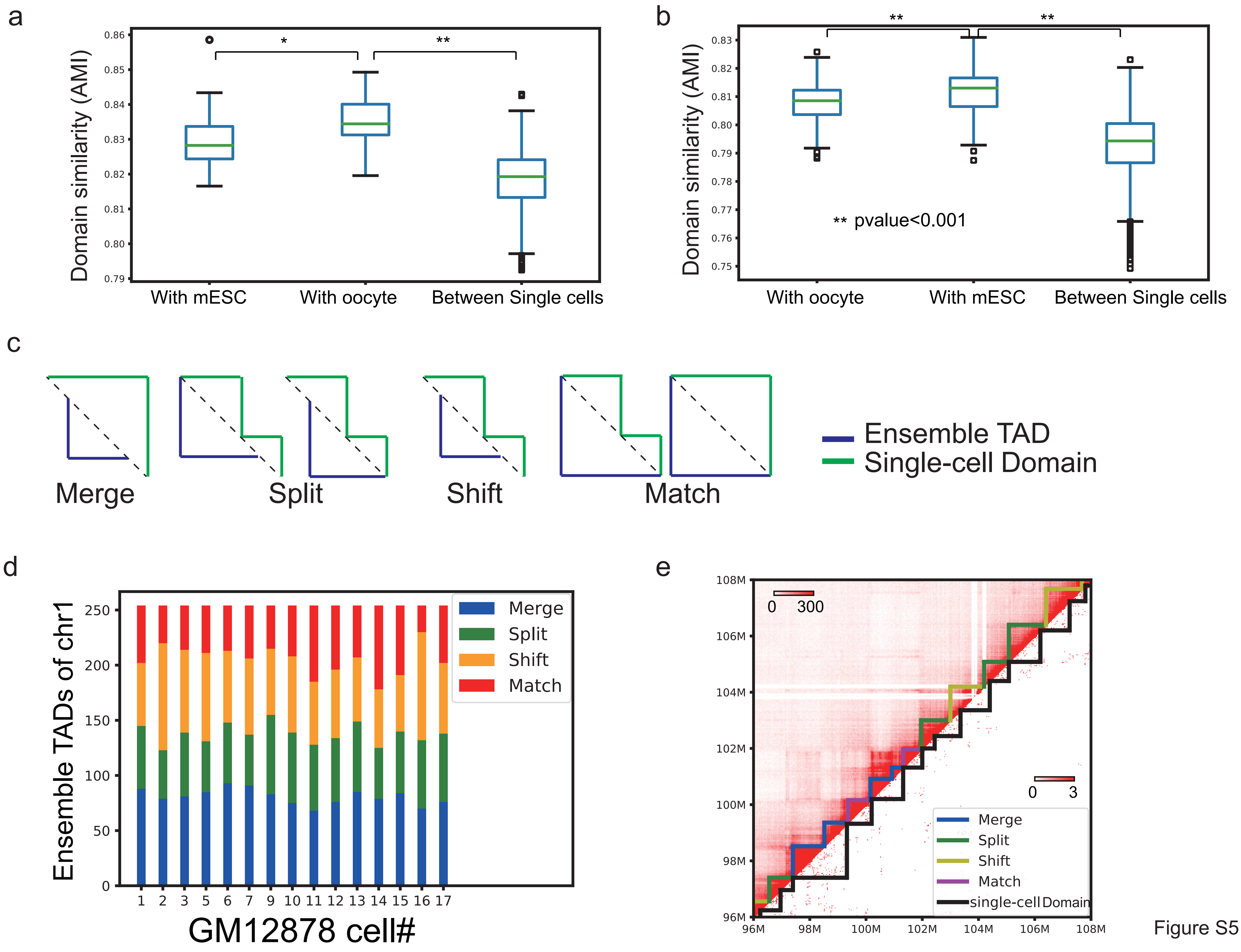
a
Domain similarity (AMI)
0.86
0.85
0.84
0.83
0.82
0.81
0.80
0.79
*
**
With mESC
With oocyte
Between Single cells
b
Domain similarity (AMI)
0.83
0.82
0.81
0.80
0.79
0.78
0.77
0.76
0.75
**
**
** pvalue<0.001
With oocyte
With mESC
Between Single cells
c
Merge
Split
Shift
Match
Ensemble TAD
Single-cell Domain
d
Ensemble TADs of chr1
250
200
150
100
50
0
Merge
Split
Shift
Match
1 2 3 5 6 7 9 10 11 12 13 14 15 16 17
GM12878 cell#
e
108M
106M
104M
102M
100M
98M
96M
96M 98M 100M 102M 104M 106M 108M
0 300
0 3
Merge
Split
Shift
Match
single-cell Domain

Figure S5

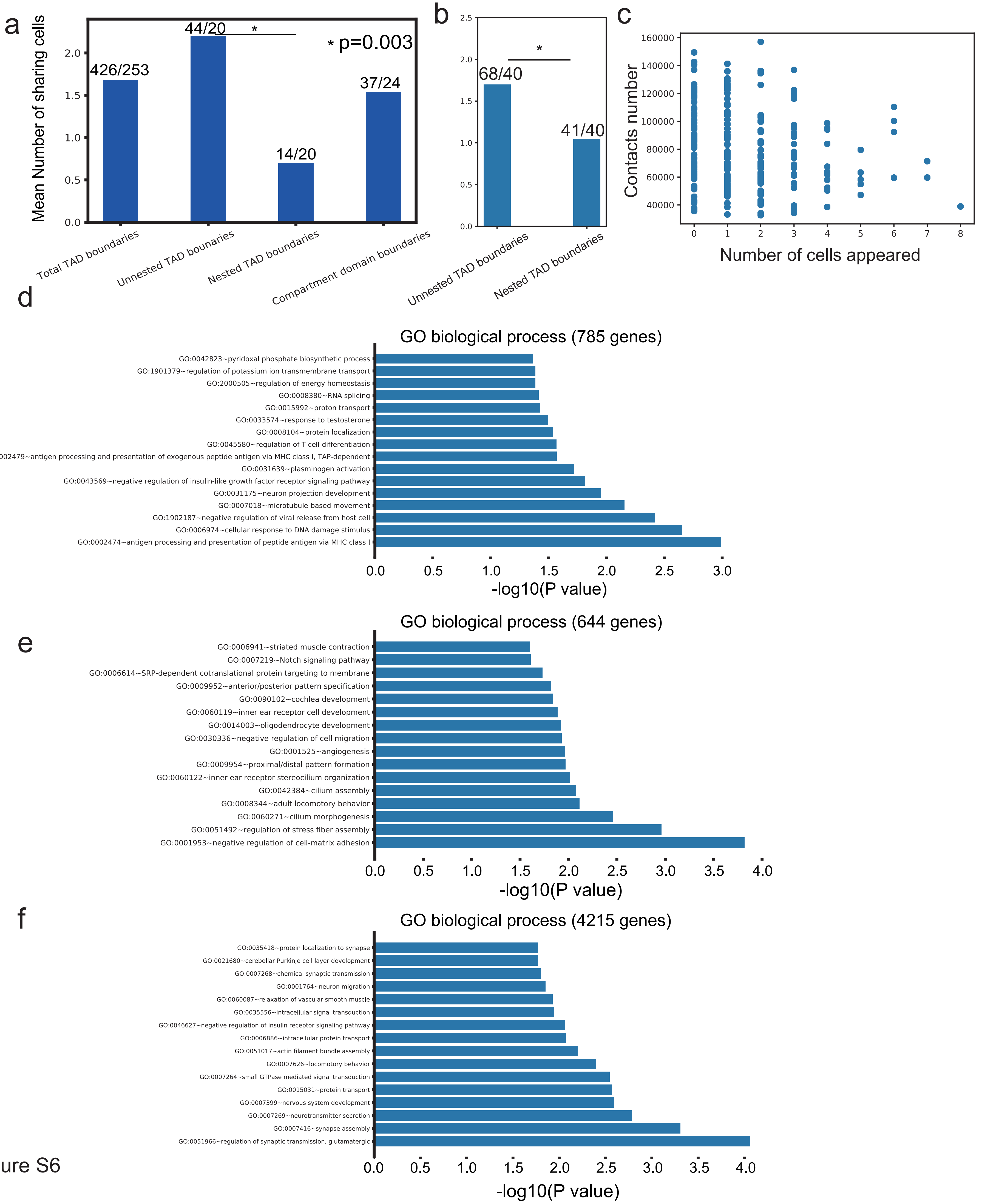

a
Mean Number of sharing cells
426/253
44/20
14/20
37/24
*
* p=0.003
0.0
0.5
1.0
1.5
2.0
Total TAD boundaries
Unnested TAD bounaries
Nested TAD boundaries
Compartment domain boundaries
b
68/40
41/40
*
0.0
0.5
1.0
1.5
2.0
2.5
Unnested TAD boundaries
Nested TAD boundaries
c
Contacts number
40000
60000
80000
100000
120000
140000
160000
0 1 2 3 4 5 6 7 8
Number of cells appeared
d
GO biological process (785 genes)
GO:0042823~pyridoxal phosphate biosynthetic process
GO:1901379~regulation of potassium ion transmembrane transport
GO:2000505~regulation of energy homeostasis
GO:0008380~RNA splicing
GO:0015992~proton transport
GO:0033574~response to testosterone
GO:0008104~protein localization
GO:0045580~regulation of T cell differentiation
GO:0002479~antigen processing and presentation of exogenous peptide antigen via MHC class I, TAP-dependent
GO:0031639~plasminogen activation
GO:0043569~negative regulation of insulin-like growth factor receptor signaling pathway
GO:0031175~neuron projection development
GO:0007018~microtubule-based movement
GO:1902187~negative regulation of viral release from host cell
GO:0006974~cellular response to DNA damage stimulus
GO:0002474~antigen processing and presentation of peptide antigen via MHC class I
0.0 0.5 1.0 1.5 2.0 2.5 3.0
-log10(P value)
e
GO biological process (644 genes)
GO:0006941~striated muscle contraction
GO:0007219~Notch signaling pathway
GO:0006614~SRP-dependent cotranslational protein targeting to membrane
GO:0009952~anterior/posterior pattern specification
GO:0090102~cochlea development
GO:0060119~inner ear receptor cell development
GO:0014003~oligodendrocyte development
GO:0030336~negative regulation of cell migration
GO:0001525~angiogenesis
GO:0009954~proximal/distal pattern formation
GO:0060122~inner ear receptor stereocilium organization
GO:0042384~cilium assembly
GO:0008344~adult locomotory behavior
GO:0060271~cilium morphogenesis
GO:0051492~regulation of stress fiber assembly
GO:0001953~negative regulation of cell-matrix adhesion
0.0 0.5 1.0 1.5 2.0 2.5 3.0 3.5 4.0
-log10(P value)
f
GO biological process (4215 genes)
GO:0035418~protein localization to synapse
GO:0021680~cerebellar Purkinje cell layer development
GO:0007268~chemical synaptic transmission
GO:0001764~neuron migration
GO:0060087~relaxation of vascular smooth muscle
GO:0035556~intracellular signal transduction
GO:0046627~negative regulation of insulin receptor signaling pathway
GO:0006886~intracellular protein transport
GO:0051017~actin filament bundle assembly
GO:0007626~locomotory behavior
GO:0007264~small GTPase mediated signal transduction
GO:0015031~protein transport
GO:0007399~nervous system development
GO:0007269~neurotransmitter secretion
GO:0007416~synapse assembly
GO:0051966~regulation of synaptic transmission, glutamatergic
0.0 0.5 1.0 1.5 2.0 2.5 3.0 3.5 4.0
-log10(P value)

Figure S6

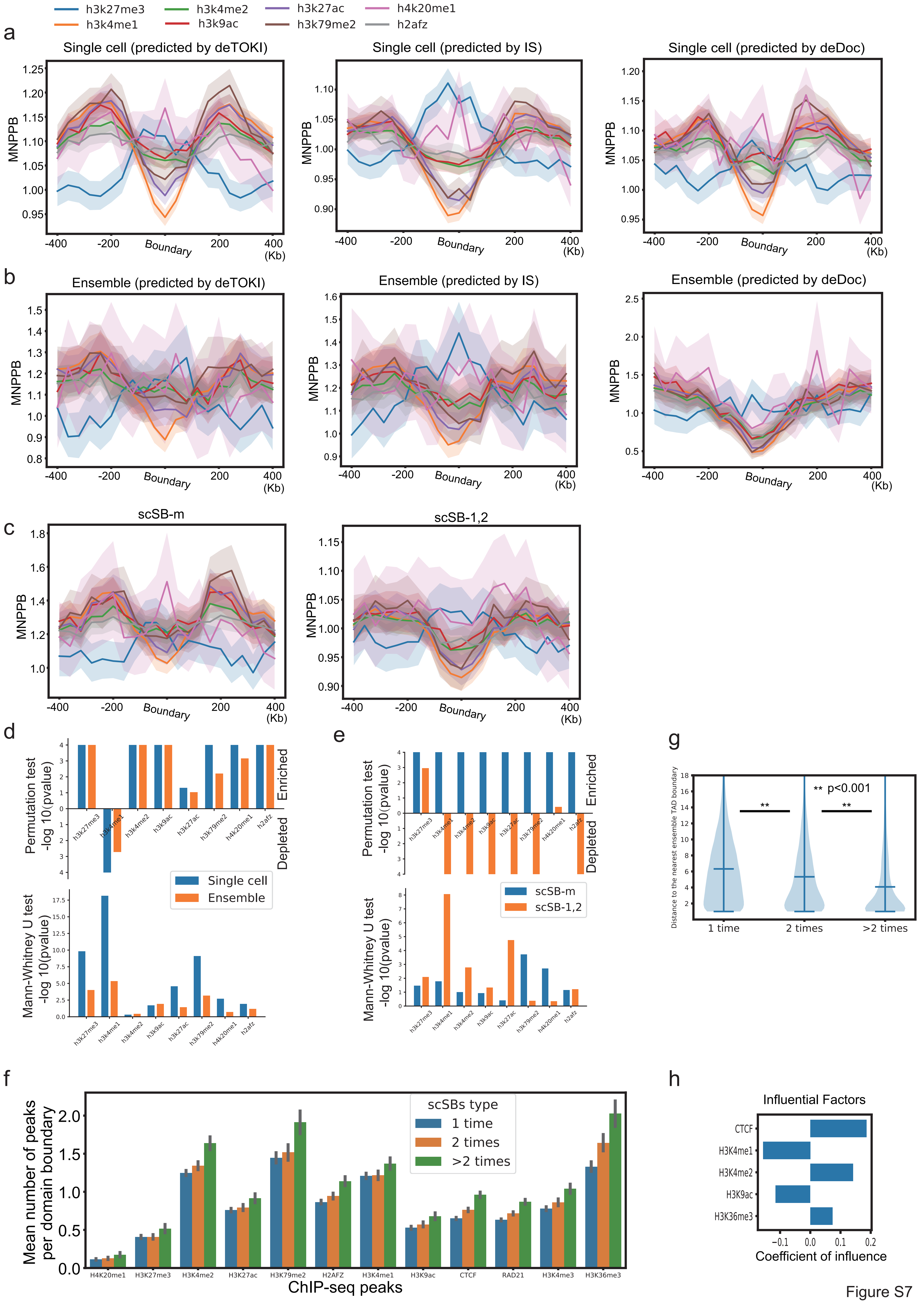
h3k27me3
h3k4me1
h3k4me2
h3k9ac
h3k27ac
h3k79me2
h4k20me1
h2afz
a
Single cell (predicted by deTOKI)
Single cell (predicted by IS)
Single cell (predicted by deDoc)
MNPPB
Boundary
(Kb)
b
Ensemble (predicted by deTOKI)
Ensemble (predicted by IS)
Ensemble (predicted by deDoc)
c
scSB-m
scSB-1,2
d
Permutation test -log 10(pvalue)
Enriched
Depleted
Single cell
Ensemble
Mann-Whitney U test -log 10(pvalue)
e
scSB-m
scSB-1,2
g
Distance to the nearest ensemble TAD boundary
** p<0.001
1 time
2 times
>2 times
f
Mean number of peaks per domain boundary
scSBs type
1 time
2 times
>2 times
H4K20me1
H3K27me3
H3K4me2
H3K27ac
H3K79me2
H2AFZ
H3K4me1
H3K9ac
CTCF
RAD21
H3K4me3
H3K36me3
ChIP-seq peaks
h
Influential Factors
CTCF
H3K4me1
H3K4me2
H3K9ac
H3K36me3
Coefficient of influence

Figure S7

a

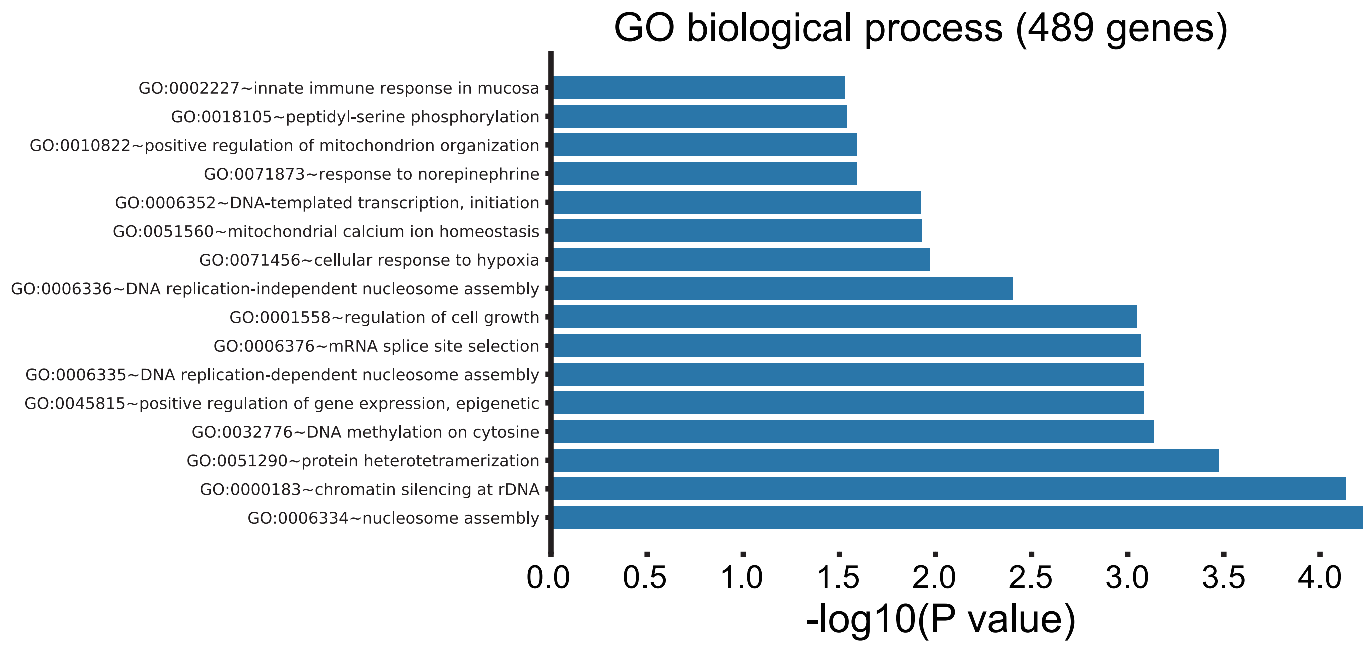

b

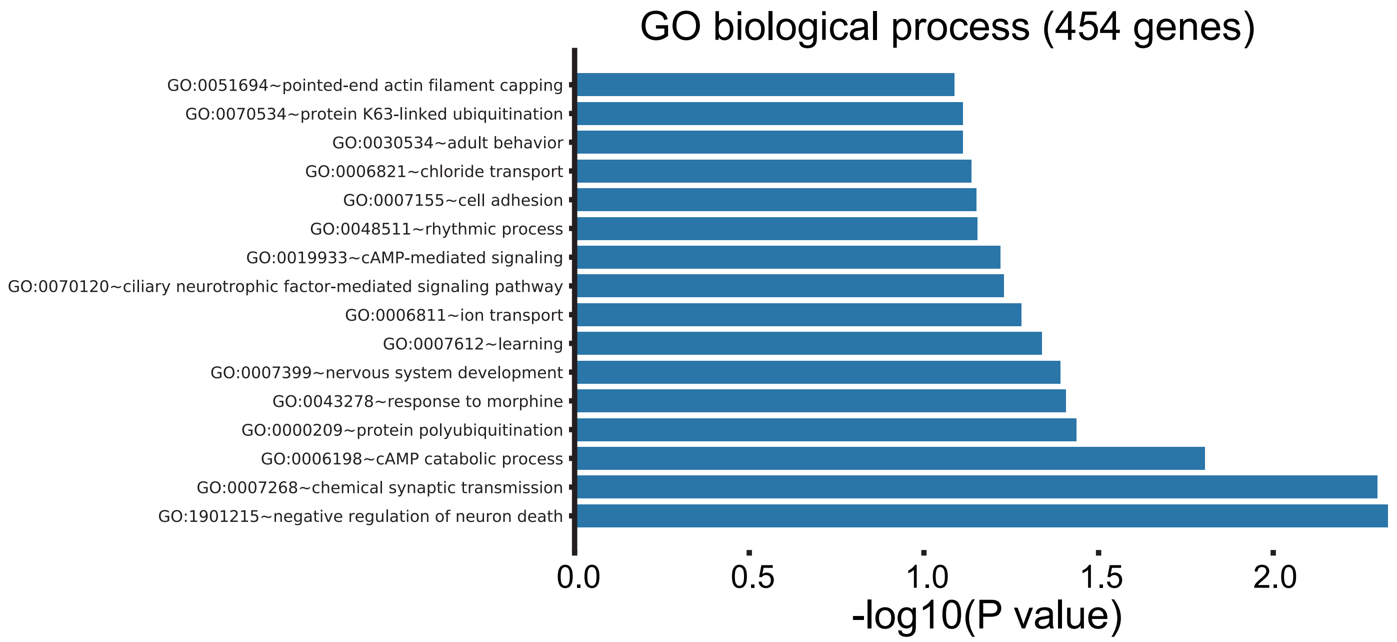

c

Chip-seq peaks	2i specific single-cell boundaries(n=346)	serum specific single-cell boundaries(n=351)	Binomial test (two-tailed)
ZC3H11A	77	95	pvalue=0.195
CHD2	33	34	pvalue=0.903
MAFK	65	64	pvalue=0.930
H3K36me3	423	546	pvalue=0.000
H3K9me3	415	341	pvalue=0.004
CTCF	181	237	pvalue=0.008
H3K4me3	118	165	pvalue=0.007
HCFC1	45	74	pvalue=0.008
ZNF384	147	165	pvalue=0.365
H3K4me1	32	15	pvalue=0.013
H3K9ac	269	345	pvalue=0.004
H3K27ac	195	206	pvalue=0.653

d

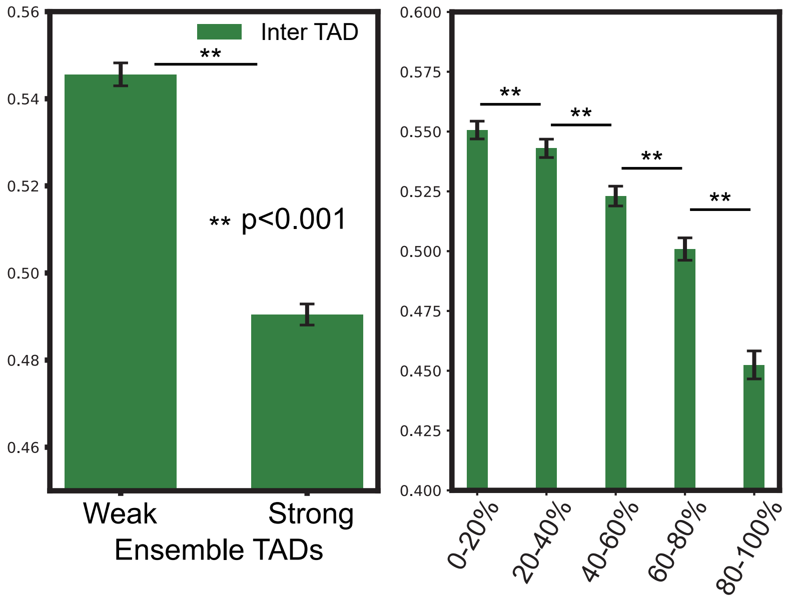

e

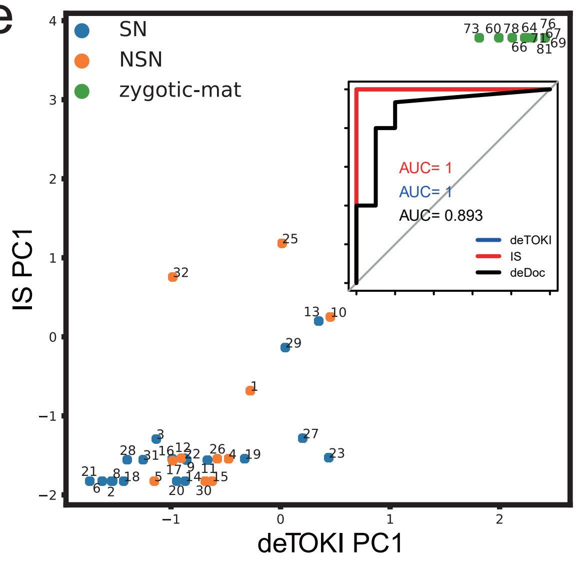

Figure S8

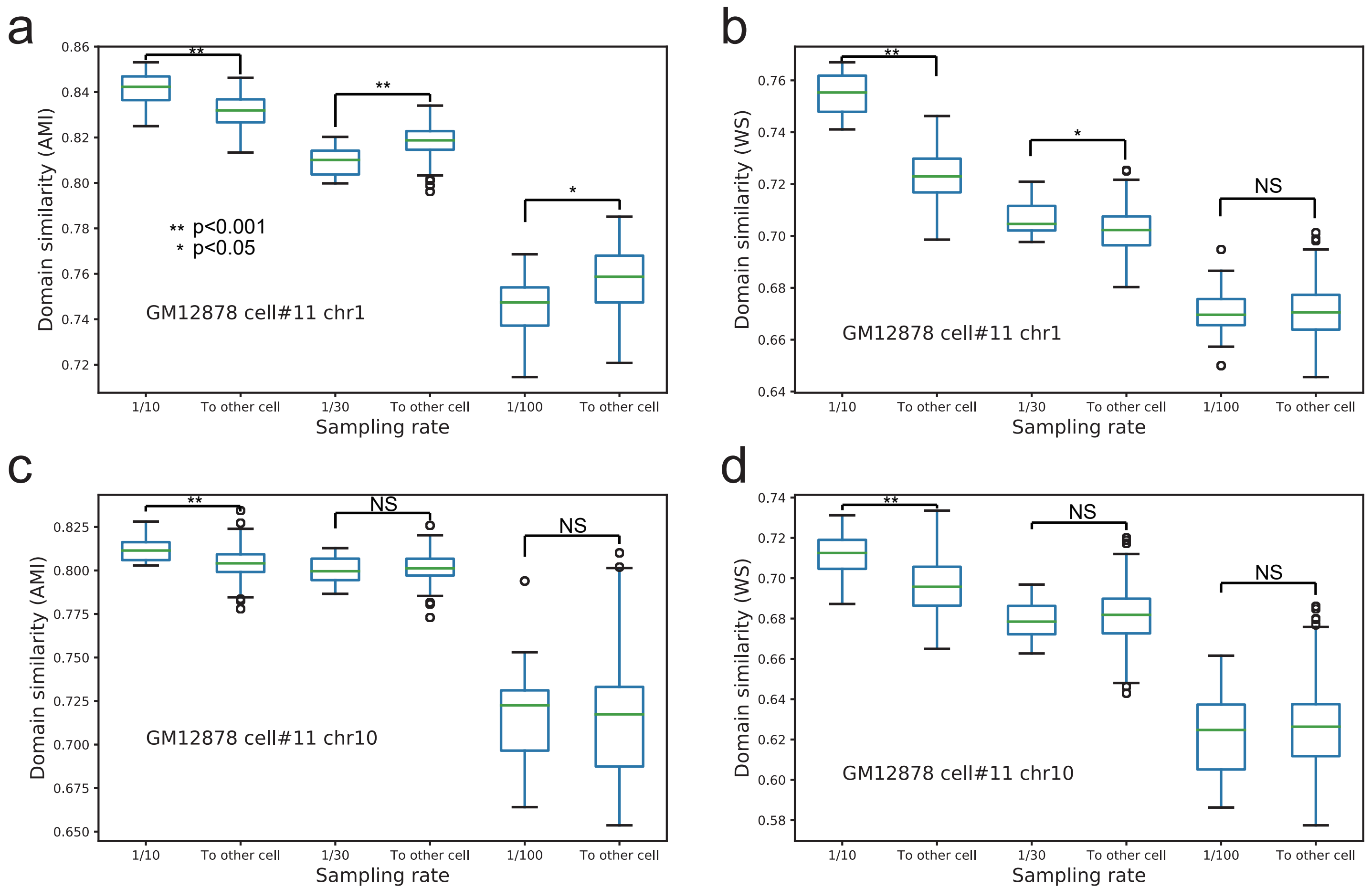
a
b
c
d
Domain similarity (AMI)
Domain similarity (WS)
Sampling rate
1/10
To other cell
1/30
1/100
** p<0.001
* p<0.05
NS
GM12878 cell#11 chr1
GM12878 cell#11 chr10
0.86
0.84
0.82
0.80
0.78
0.76
0.74
0.72
0.70
0.68
0.66
0.64
0.825
0.800
0.775
0.750
0.725
0.700
0.675
0.650
0.62
0.60
0.58

Figure S9

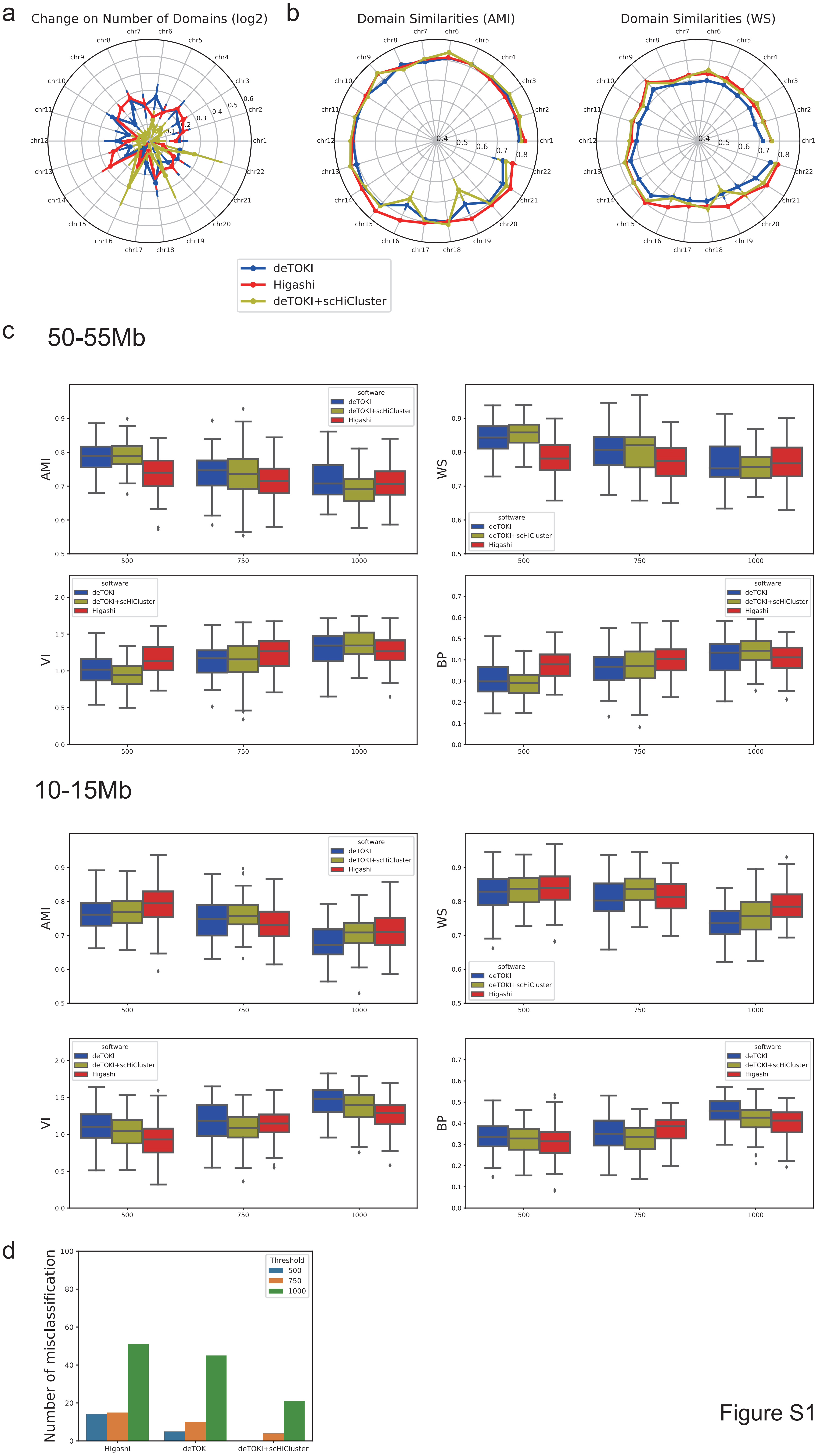
a
Change on Number of Domains (log2)
b
Domain Similarities (AMI)
Domain Similarities (WS)
chr1
chr2
chr3
chr4
chr5
chr6
chr7
chr8
chr9
chr10
chr11
chr12
chr13
chr14
chr15
chr16
chr17
chr18
chr19
chr20
chr21
chr22
deTOKI
Higashi
deTOKI+scHiCluster
c
50-55Mb
10-15Mb
software
deTOKI
deTOKI+scHiCluster
Higashi
AMI
WS
VI
BP
500
750
1000
d
Number of misclassification
Threshold
500
750
1000
Higashi
deTOKI
deTOKI+scHiCluster

Figure S10
