# Supplementary material for: DeTOKI identifies and characterizes the dynamics of chromatin TAD-like domains in a single cell
Source: Genome Biol. 2021 Jul 27;22:217. doi: 10.1186/s13059-021-02435-7 (PMC8314462; doi:10.1186/s13059-021-02435-7)
Supplement: Supplementary file 6 — Additional file 6: Table S4. CPU/GPU running times with single cell Hi-C of GM12878 cells in Tan’s data. [file 13059_2021_2435_MOESM6_ESM.docx]

Table. run times of tools used for calling TAD of GM12878 single cell Hi-C in [Tan et al.].

| tools | deTOKI | IS | deDoc | GRiNCH | SpectralTAD | schicluster | Higashi（1gpu） |
| --- | --- | --- | --- | --- | --- | --- | --- |
| chr1 | 108.53s | 873.46s | 9.40s | 49.44s | 130.41s | 269.90s | 320min |
| chr2 | 110.92s | 810.36s | 9.97s | 48.66s | 112.83s | 273.20s | 295min |
| chr3 | 87.14s | 442.47s | 8.81s | 49.88s | 81.49s | 193.77s | 292min |
| chr4 | 87.94s | 399.11s | 8.19s | 33.33s | 78.80s | 181.33s | 300min |
| chr5 | 78.31s | 339.92s | 7.51s | 36.08s | 78.80s | 160.14s | 433min |
| chr6 | 71.85s | 290.92s | 7.05s | 33.44s | 73.92s | 137.10s | 276min |
| chr7 | 65.99s | 235.72s | 6.37s | 32.58s | 65.16s | 111.69s | 353min |
| chr8 | 60.99s | 184.92s | 6.00s | 27.85s | 58.81s | 92.37s | 271min |
| chr9 | 49.84s | 166.98s | 4.30s | 21.15s | 52.50s | 63.72s | 391min |
| chr10 | 55.62s | 151.32s | 5.03s | 24.43s | 50.13s | 78.89s | 275min |
| chr11 | 54.54s | 149.15s | 4.81s | 23.42s | 50.62s | 75.46s | 286min |
| chr12 | 55.00s | 144.14s | 5.03s | 22.83s | 50.40s | 75.19s | 281min |
| chr13 | 42.23s | 93.55s | 3.86s | 16.07s | 40.99s | 41.94s | 409min |
| chr14 | 37.21s | 76.41s | 3.65s | 13.50s | 37.08s | 34.97s | 238min |
| chr15 | 32.38s | 67.11s | 2.93s | 10.12s | 35.05s | 27.44s | 272min |
| chr16 | 31.94s | 47.04s | 2.93s | 9.44s | 30.23s | 24.72s | 275min |
| chr17 | 29.47s | 34.85s | 3.04s | 7.58s | 27.62s | 23.66s | 462min |
| chr18 | 30.80s | 31.19s | 3.09s | 7.45s | 25.74s | 21.77s | 282min |
| chr19 | 22.16s | 14.79s | 2.54s | 3.66s | 19.99s | 11.68s | 243min |
| chr20 | 23.91s | 17.46s | 2.66s | 4.25s | 20.99s | 13.45s | 487min |
| chr21 | 16.17s | 8.60s | 1.71s | 2.36s | 17.30s | 5.94s | 262min |
| chr22 | 15.51s | 10.20s | 1.64s | 2.54s | 17.73s | 6.03s | 260min |
| chrX | 67.12s | 219.89s | 6.69s | 27.95s | 62.92s | 115.14s | 420min |

Note: The value is the mean run time on one cell’s data. run time of Higashi is test on cell 2.

CPU: Intel(R) Xeon(R) Gold 6240 CPU @ 2.60GHz

GPU: Tesla V100 SXM2 32GB
