# Supplementary material for: DeTOKI identifies and characterizes the dynamics of chromatin TAD-like domains in a single cell
Source: Genome Biol. 2021 Jul 27;22:217. doi: 10.1186/s13059-021-02435-7 (PMC8314462; doi:10.1186/s13059-021-02435-7)
Supplement: Supplementary file 8 — Additional file 8: Review history. [file 13059_2021_2435_MOESM8_ESM.docx]

Review History

**First round of review**

**Reviewer 1**

**Were you able to assess all statistics in the manuscript, including the appropriateness of statistical tests used?**

Yes, and I have assessed the statistics in my report.

**Were you able to directly test the methods?**

No

**Comments to author:**

Li and Zhang present an interesting manuscript describing deTOKI, a method for identifying Topologically Associating Domains (TADs) from sparse Hi-C data, with the most relevant application being analysis of TAD structure in single cell Hi-C data. The method uses NMF to identify TADs from the sparse contact maps. The authors perform several analysis using simulated data to validate the accuracy and the tool. They also apply this to analyze publicly available single-cell Hi-C data and come to some quite interesting conclusions. Some of these (that single-cell TAD structure is highly variable between cells but in aggregate agree with ensemble measurements) is expected and has been seen before using imaging methods. They also have some additional analysis of boundaries that are under or over-represented in single cells compared to ensemble and their relationship with epigenetic marks. This analysis to the best of my knowledge is quite novel, and it makes interesting predictions about the function of these single-cell specific boundaries (or highly shared boundaries). All in all, I think that this is a good manuscript, and most of the comments that I have are minor. I think that with minor revisions this study will be appropriate for publication in genome biology.

Specific comments:

First, and this is mostly a complement more than anything else, I really like the way they do the subsampling. In particularly using the simulated 3D models of chromatin. This gets over some serious challenges in generating simulated single-cell Hi-C data for tool development. I think it would be valuable for them to include/publish these methods along with the manuscript. I checked the github repo and it seems like is mostly just the deTOKI code, but I think that this simulated method is also quite useful.

Related to the prior comment, they perform sub-sampling to depths consistent with the Flyamer single-cell Hi-C data. This is nice, but the Flyamer data is still relatively high-coverage single cell Hi-C data. I think they should include additional subsampling to even lower depth. For example, the Ramani sci-HiC data is more than an order of magnitude lower in contacts/cell. This is obviously quite sparse, but the value is that these methods can be performed on larger numbers of cells. In this sense I think it is valuable to know if deTOKI works well in even sparser data. This will be useful for helping experimental scientists determine optimal experimental designs when generating single-cell Hi-C data.

Several recent methods - scHiCluster and Higashi - employ methods for "imputing" missing contacts in single cell Hi-C data. How does this affect predictions based on deTOKI?

They mention this in the discussion and methods, but I think it is worthwhile to have a somewhat more in depth analysis of run times of the methods compared to other tools. For instance, they say that it took 1 hour to run on Flyamer's data at 40kb resolution. Is this on all the data? A single cell? A single chromosome?

There are a few language issues that I think would be helpful to address:

Throughout the manuscript, they refer to the structures in single cells as "TADs". However, I think it is hard to say that they are "TADs" and not something like "compartment domains" (see Beagan and Cremins, Nature Genetics 2020 for an excellent review on this). I think it is worthwhile to at least mention that the tool will not really be able to distinguish the mechanisms that generate these "domain like structures" in single cells (i.e. CTCF/Cohesin vs. compartmentalization). This can be a contentious area in the field.

In the introduction, they state: "However, by applying deTOKI to public experimental

single-cell Hi-C data, we found that although the TAD structure is highly dynamic between the cells, the TADs adhere to the ensemble, suggesting their tight regulation." By the end of the paper, I understood what they meant, but I found this sentence somewhat confusing in the introduction. If they can re-phrase it to clarify their findings I think that would be helpful.

I think the authors should give themselves a bit more credit than saying their method is "fine" in the following sentence: "Taken together, our assessment suggests that deTOKI works fine with experimental single-cell Hi-C data." The tool seems to work "well" by my estimation.

Some labelling issues with figures:

-No units associated with Figure 4a.

-Supplemental Figure 5e - the "match" labels are in red so it is very hard to see these against the red coloring of the heatmap.

**Reviewer 2**

**Were you able to assess all statistics in the manuscript, including the appropriateness of statistical tests used?**

No, I do not feel adequately qualified to assess the statistics.

**Were you able to directly test the methods?**

No

**Comments to author:**

Summary

deTOKI is a new method for identifying TADs even from an ultra-low resolution HiC matrix, which makes it suitable for analyzing single cell HiC experiments (scHiC). Algorithmically, it utilizes a divide-and-conquer approach by splitting the chromosome into 8Mb bins and then using NMF to cluster bins and finally refining the position of the TAD boundaries by finding local maxima of the insulation score.

To validate and compare their method, the authors employed 2 techniques. First, they downsampled ensemble HiC data and checked how robust were each method's predictions. Second, they generated a scHiC matrix from simulated 5Mb chromatin structure and checked how well each method could reproduce the known architecture.

By applying their method to publicly available single cell datasets, they analyzed the differences between TAD boundaries in the ensemble vs single cell cases. They found that there is high variation in the number of ensemble boundaries utilized by single cells and also a significant number of single-cell specific boundaries (scSB). They characterize these boundaries by running several enrichment analyses to investigate their structural as well as functional characteristics.

Comments

Lack of clarity

Some parts of the paper are not explained adequately which may affect the reproducibility of the research. In particular:

With regards to the algorithm:

*	They do not explain how they deal with TADs at the boundary of their 4Mb window.

*	It's unclear how 2 bins cluster together (lines 17-19, p.27). What does "raw" refer to?

*	What window to they use to define "local" minimum/maximum.

*	The definition of bin strength is confusing. Maybe in line 8 p.28 they mean "local minimum"?

*	They don't explain how they performed the complexity analysis. The figures in Fig. S1 are not labeled and the units of the y-axis is unclear (it looks like it's second but this doesn't match the statements in the main text).

*	Radar plots are hard to read. They should consider standard barplots with error bars or boxplots

With regards to the analysis:

Figure 2d is unreadable. The authors should group them closer together and space out the thresholds.

How did they test for binomial fit (at p.16 line 7: P < 0.001)

For the GO enrichment analysis of popular and unpopular boundaries. They don't explain which genes they included ("genes close to the boundary"). How close? Why not all the genes in the TAD?

Figure 4c is confusing. + it's not clear how they calculated the expected counts ("permutation control") + if the x-axis is showing the scSB classes shouldn't the label be "# of cells"? + it's unclear what the y-axis refers to. Why "bins" and not "boundaries"?

Figures 4d-e and 4f-g are hard to read and validate their claims. + The y-scales differ and the lines are barely distinguishable. They should have a panel for each histone mark with 2 lines for those that differ between conditions. + Perhaps they should consider using a package like Deeptools if it's applicable.

It's unclear how they calculated the correlation of methylation pattern between inter and intra TAD bins. - how did they align the bins - how did the separate TAD boundaries into weak and strong (thresholds). - it would be interesting to see a figure of correlation or difference in methylation level as a function of TAD boundary.

Algorithm Comparisons

The authors only compared their method with methods developed for ensemble HiC. They should consider including some methods specifically developed for single cell TADs like scHiCluster.

TADs have a hierarchical/nested structure. The author should discuss how their method deals with this. It seems that by exploring the structure of the coefficient matrix and/or the search for local maxima of strength they could tune the result to different resolutions.

Have they experimented with using k-means or modularity clustering instead of NMF? k-means can be thought of as a form of NMF and it could speed up the process. It also eliminates the need of using a heuristic to assign bins to clusters.

Issues with the analysis.

In addition to the comments about the readability of the figures that make the claims hard to validate.

It is unclear what is the significance of the enrichment analysis of popular vs unpopular boundaries. If they want to claim that these boundaries have a functional role they should also include a GO analysis for the other (middle of the pack) TADs. If they want to claim that the distribution is non-random vs the scSB then they should show that these are not enriched in any term.

The definition of "unnested" TADs is not intuitive nor present in the reference. Moreover, they do not provide amble justification for the thresholds they used. They should show a plot of the strength of cross-boundary interactions vs the number of cells per TAD to demonstrate the overall relationship of the 2.

The conclusion (lines 3-6 in p. 18) is not obvious from the figure 4c. The figure shows a steady drop in the number of bins/boundaries so why "few bins were taken as boundary in only one or two cells"?

MNPPB is unnormalized with respect to the number of peaks in each dataset. Does this affect the comparison?

Probable Typos

*	paragraph from p.6 line 21 to p.7 line 13: they probably mean "i.e." instead of "e.g."

*	page 30: the term N is used before it's defined.

*	page 32: the denominator of T_{i,j} should probably be sum |S_ik * Sjk| otherwise T_{i,j} = 1.

**Authors Response**

**Point-by-point responses to the reviewers’ comments:**

Reviewer reports:

Reviewer #1:

Li and Zhang present an interesting manuscript describing deTOKI, a method for identifying Topologically Associating Domains (TADs) from sparse Hi-C data, with the most relevant application being analysis of TAD structure in single cell Hi-C data. The method uses NMF to identify TADs from the sparse contact maps. The authors perform several analysis using simulated data to validate the accuracy and the tool. They also apply this to analyze publicly available single-cell Hi-C data and come to some quite interesting conclusions. Some of these (that single-cell TAD structure is highly variable between cells but in aggregate agree with ensemble measurements) is expected and has been seen before using imaging methods. They also have some additional analysis of boundaries that are under or over-represented in single cells compared to ensemble and their relationship with epigenetic marks. This analysis to the best of my knowledge is quite novel, and it makes interesting predictions about the function of these single-cell specific boundaries (or highly shared boundaries). All in all, I think that this is a good manuscript, and most of the comments that I have are minor. I think that with minor revisions this study will be appropriate for publication in genome biology.

Specific comments:

First, and this is mostly a complement more than anything else, I really like the way they do the subsampling. In particularly using the simulated 3D models of chromatin. This gets over some serious challenges in generating simulated single-cell Hi-C data for tool development. I think it would be valuable for them to include/publish these methods along with the manuscript. I checked the github repo and it seems like is mostly just the deTOKI code, but I think that this simulated method is also quite useful.

*Answer: Thanks for the comment. We have uploaded the code for single-cell Hi-C simulation to the github repo at https://github.com/lixiaoms/TOKI.*

Related to the prior comment, they perform sub-sampling to depths consistent with the Flyamer single-cell Hi-C data. This is nice, but the Flyamer data is still relatively high-coverage single cell Hi-C data. I think they should include additional subsampling to even lower depth. For example, the Ramani sci-HiC data is more than an order of magnitude lower in contacts/cell. This is obviously quite sparse, but the value is that these methods can be performed on larger numbers of cells. In this sense I think it is valuable to know if deTOKI works well in even sparser data. This will be useful for helping experimental scientists determine optimal experimental designs when generating single-cell Hi-C data.

*Answer: Thanks for the comment.*

*We have performed additional assessment. The deTOKI code can be run with any amount of data. Therefore, the more salient question is at what data coverage level does the deTOKI predicted domain become unreliable. We have shown that deTOKI can precisely predict domains from the data of Tan et al., which have the highest data coverage of all published single-cell Hi-C data. Therefore, we performed subsampling from the data of GM12878 cell #11 in this dataset. Data coverage of the subsample was regarded sufficient, as long as the similarity between the domains predicted from the subsample and full data of cell #11 remains significantly larger than that between subsample and the data of other cells. Otherwise, data coverage was regarded insufficient. As shown in the figure (see PDF version), added to the revised manuscript as Supplementary Fig. S9, when sampling rates were above 1/10, i.e., more than 0.1M contacts per cell, then the predicted domains were reliable. However, when rates fell below 1/30, i.e., less than 0.04M contact per cell, coverage was considered insufficient. Therefore, for most of the cells in Ramani’s dataset that have about 0.01M contacts per cell, deTOKI will not work. We have added a new section in the supplemental text to address this issue.*

Several recent methods - scHiCluster and Higashi - employ methods for "imputing" missing contacts in single cell Hi-C data. How does this affect predictions based on deTOKI?

*Answer: Thanks for the comment.*

*We did such assessment and found deTOKI to be more efficient on TAD-like domain identification with single-cell Hi-C data in terms of CPU running time. We added a new section in the supplementary text to address this issue, as copied below.*

*“To assess the improvement that data imputation may introduce to TAD-like domain predictions, we compared the performance of deTOKI, deTOKI with data imputation by scHiCluster, denoted as deTOKI+scHiCluster, and Higashi.*

*First, we compared the three tools with the downsampled 1/800 data of Dixon et al. and found comparable performance among the three. The largest absolute log2 fold changes (|log2FC|) in predicted TAD-like domain numbers between the downsampled datasets were all less than 0.3, and deTOKI+scHiCluster had the lowest |log2FC| on average (Supplementary Fig.10a) Taking the TADs identified with the full data as the gold standard, we next compared the adjusted mutual information (AMI) and weighted similarity (WS) among the tools. All three tools showed values of AMI and WS rather close to each other with AMIs all around 0.8 on average and WS about 0.71 (deTOKI+scHiCluster and Higashi were slightly better with average WS=0.74, Supplementary Fig.10b).*

*Second, we compared the three tools with simulated single-cell Hi-C data with setting identical to that in the main text between the genome region of chr18:50-55Mb and 10-15Mb (Supplementary Fig.10c). For all four indices assessed (AMI, WS, VI and BP), we found that the three tools performed in a similar manner. In the region chr18: 50-55Mb, deTOKI and deTOKI+scHiCluster had better performance on all four indices than Higashi. In the region chr18: 10-15Mb, Higashi and deTOKI+scHiCluster had better performance than deTOKI alone in about half the settings. Last, for the classification task, deTOKI+scHiCluster had the lowest misclassification number compared to Higashi or deTOKI alone (Supplementary Fig.10d).*

*Taken together, deTOKI and Higashi had similar performance, and deTOKI could be further improved by data imputation, e.g., scHiCluster. Considering the substantial CPU time required by Higashi (about 100-fold more CPU time required by either deTOKI or scHiCluster, Supplementary Table5), deTOKI is more efficient on TAD-like domain identification with single-cell Hi-C data.”*

They mention this in the discussion and methods, but I think it is worthwhile to have a somewhat more in depth analysis of run times of the methods compared to other tools. For instance, they say that it took 1 hour to run on Flyamer's data at 40kb resolution. Is this on all the data? A single cell? A single chromosome?

*Answer: Thanks for the comment.*

*In the legend of Figure S1C, we further explained that each data point represents an intra-chromosome Hi-C contact matrix from oocyte ensemble Hi-C data. Therefore, an hour here refers to the run time for the whole ensemble Hi-C data. We also systematically assess the run times for all tools compared. The data we used were from Tan et al., and the assessment was performed on a laptop with CPU: Intel(R) Xeon(R) Gold 6240 CPU @ 2.60GHz. We ran the tools in each chromosome individually, recorded the run times, and summarized into Supplementary Table S5, see PDF version.*

There are a few language issues that I think would be helpful to address:

Throughout the manuscript, they refer to the structures in single cells as "TADs". However, I think it is hard to say that they are "TADs" and not something like "compartment domains" (see Beagan and Cremins, Nature Genetics 2020 for an excellent review on this). I think it is worthwhile to at least mention that the tool will not really be able to distinguish the mechanisms that generate these "domain like structures" in single cells (i.e. CTCF/Cohesin vs. compartmentalization). This can be a contentious area in the field.

*Answer: Thanks for the comment.*

*We agree with the reviewer, and we have used the term “TAD-like domain” or “domain” throughout the revised text.*

In the introduction, they state: "However, by applying deTOKI to public experimental single-cell Hi-C data, we found that although the TAD structure is highly dynamic between the cells, the TADs adhere to the ensemble, suggesting their tight regulation." By the end of the paper, I understood what they meant, but I found this sentence somewhat confusing in the introduction. If they can re-phrase it to clarify their findings I think that would be helpful.

*Answer: Thanks for the comment.*

*We have rewritten the paragraph to read as follows:*

*“We present evidence that deTOKI can reliably predict TAD-like domain structures at the single-cell level. Although TAD-like domain structures are highly dynamic between cells, they are not randomly distributed in the cell population, implying the existence of tight regulation on the domains at the single-cell level.”*

I think the authors should give themselves a bit more credit than saying their method is "fine" in the following sentence: "Taken together, our assessment suggests that deTOKI works fine with experimental single-cell Hi-C data." The tool seems to work "well" by my estimation.

*Answer: We appreciate this comment, and following this suggestion, we have exchanged “fine” for “well”.*

Some labelling issues with figures:

-No units associated with Figure 4a.

*Answer: Thanks for pointing out this issue; we have put the units into the figure.*

-Supplemental Figure 5e - the "match" labels are in red so it is very hard to see these against the red coloring of the heatmap.

*Answer: Thanks for the suggestion. We have now drawn the plot with a better color contrast.*

Reviewer #2: Summary

deTOKI is a new method for identifying TADs even from an ultra-low resolution HiC matrix, which makes it suitable for analyzing single cell HiC experiments (scHiC). Algorithmically, it utilizes a divide-and-conquer approach by splitting the chromosome into 8Mb bins and then using NMF to cluster bins and finally refining the position of the TAD boundaries by finding local maxima of the insulation score.

To validate and compare their method, the authors employed 2 techniques. First, they downsampled ensemble HiC data and checked how robust were each method's predictions. Second, they generated a scHiC matrix from simulated 5Mb chromatin structure and checked how well each method could reproduce the known architecture.

By applying their method to publicly available single cell datasets, they analyzed the differences between TAD boundaries in the ensemble vs single cell cases. They found that there is high variation in the number of ensemble boundaries utilized by single cells and also a significant number of single-cell specific boundaries (scSB). They characterize these boundaries by running several enrichment analyses to investigate their structural as well as functional characteristics.

Comments

Lack of clarity

Some parts of the paper are not explained adequately which may affect the reproducibility of the research. In particular:

With regards to the algorithm:

*They do not explain how they deal with TADs at the boundary of their 4Mb window.

*Answer: Thanks for the comment.*

*To address our handling of sliding windows, any given window can be separated into four bins. For example, a window with size=8Mb can be separated into Bin1, Bin2, Bin3, and Bin4 with size=2Mb each. As Bin1 and Bin2 also belong to the 5’upstream window, Bin3 and Bin4 also belong to the 3’downstream window. Since Bin1 and Bin4 will be more affected by the two flanking windows, we only take the boundaries predicted in Bin2 and Bin3 as the output of deTOKI. This strategy can be applied to any window size with Bin size = window size / 4. We have added these details to the revised main text.*

*It's unclear how 2 bins cluster together (lines 17-19, p.27). What does "raw" refer to?

*Answer: Thanks for the comment.*

*We apologize for the typo, and “raw” is changed to “row”. We cluster the bins according to a row’s maximum coordinate in each column of the coefficient matrix, which means that bin i and bin j are clustered if the maximums in column i and j of the coefficient matrix are in the same row.*

*What window to they use to define "local" minimum/maximum.

*Answer: Thanks for the comment. The window is 200 bins with binsize=40kb, according to the newly added formula where*

*“CRi is called the local minimum cluster rate if the following inequality is satisfied:*

*∑_(j=i)^(i+2)▒〖CR〗_j -∑_(j=i-3)^(i-1)▒〖〖CR〗_j<0≤〗 ∑_(j=i+1)^(i+3)▒〖CR〗_j -∑_(j=i-2)^i▒〖CR〗_j .” (see PDF version for better format)*

*The definition of bin strength is confusing. Maybe in line 8 p.28 they mean "local minimum"?

*Answer: Thanks for the comment.*

*We added two formulas in Methods to formally define the bin strength and local minimum. The concepts were rather straightforward with the help from Figure 1.d. The local minimums were the curve valley bottoms in the cluster rate curve, while the bin strength is the max height of the flanking peaks, i.e., top minus bottom. We copied the two formulas below, please go to PDF version for better format.*

*“CRi is called the local minimum cluster rate if the following inequality is satisfied: (see PDF version)*

*∑_(j=i)^(i+2)▒〖CR〗_j -∑_(j=i-3)^(i-1)▒〖〖CR〗_j<0≤〗 ∑_(j=i+1)^(i+3)▒〖CR〗_j -∑_(j=i-2)^i▒〖CR〗_j .*

*The location and strength of the bins that have local minimum cluster rates were recorded. We assume the location set is {i_1,i_2,…,i_m }. For any t=1...m, the strength of 〖bin〗_(i_t ) was defined as the local maximum CR minus 〖CR〗_(i_t ).*

*Strength of 〖bin〗_(i_t )=max{〖CR〗_(i_(t-1) ),〖CR〗_(i_(t-1)+1),…,〖CR〗_(i_(t+1) ) }-〖CR〗_(i_t ),*

*where i0 = 1, im+1 = 200.”*

*They don't explain how they performed the complexity analysis. The figures in Fig. S1 are not labeled and the units of the y-axis is unclear (it looks like it's second but this doesn't match the statements in the main text).

*Answer: Thanks for the comment. We added a paragraph in Methods to clarify the complexity analysis and labeled chromosome numbers in Supplementary Figure 1c (see PDF version).*

*As deTOKI runs on each sliding window independently, the overall complexity can be written as O(n) where n represents the number of sliding windows for the whole genome. This complexity can also be evidenced by the almost linear correlation with chromosome lengths (Supplementary Figure 1c). Within each window, let the window size be L and binsize be B; then we have the contact matrix size = L/B. Because the basic computing unit in deTOKI is NMF, which has the complexity of O(ab), where a and b represent the number of rows and columns in the input matrix, i.e., the contact matrix, the complexity for each NMF is O((L/B)2). Together, the total computing complexity is O(n*(L/B)2).*

*The units of Y-axis in Figure S1c is second, and the running time for Flyamer’s ensemble Hi-C data can be obtained by summing up the times from all chromosomes. In our test, it took deTOKI about one hour on a single-core CPU and about 6 minutes with 16 cores to complete the job on Flyamer’s data.*

*Radar plots are hard to read. They should consider standard barplots with error bars or boxplots

*Answer: Thanks for the comment.*

*We compared the radar plot vs. standard barplot and found that the standard barplot will take the whole row in a page, making it hard to fit other panels in the figure (see example below). Moreover, the radar plot provides simple visual guidance showing that deTOKI performed better than other tools by keeping within inner (Fig. 2a and see PDF version) or outer (Fig. 2b and see PDF version) circles. We agreed with the reviewer that the annotations of radar plot in our previous version was not perfect, and we reannotated the plot to make it clear. We also added the error bars to the radar plots.*

With regards to the analysis:

Figure 2d is unreadable. The authors should group them closer together and space out the thresholds.

*Answer: Thanks for the comment. We adjusted the distance between groups and spaced out the thresholds as the reviewer suggested.*

How did they test for binomial fit (at p.16 line 7: P < 0.001) For the GO enrichment analysis of popular and unpopular boundaries. They don't explain which genes they included ("genes close to the boundary"). How close? Why not all the genes in the TAD?

*Answer: Thanks for the comment.*

*In the binomial test, the null hypothesis is that the number of ensemble TAD boundaries appearing in the individual cells as the TAD-like domain boundary follows a binomial distribution. We chose Li’s data as an example and modeled the distribution with a binomial B(150,0.06), where 150 is the number of cells and 0.06 is the average frequency with which an ensemble TAD boundary appears in a single cell. We found that 453 and 452 boundaries (out of 2602) appeared in more than 12 and in less than 6 cells, respectively (Fig.4b). By a python function scipy.stats.binom_test (452, n=2602, p=0.108, alternative='two-sided') and scipy.stats.binom_test (453, n=2602, p=0.117, alternative='two-sided'), we got P< 0.001, which rejected the null hypothesis that the number follows a binomial distribution.*

*The genes in the GO analysis were those that have at least one bp overlapped with the TAD boundaries, i.e., the bin in 40kb resolution. To make it clear, we changed the text from “genes close to the boundary” to “genes on the boundary”. We didn’t include all the genes in the TADs because the two boundaries of an ensemble TAD may belong to popular and unpopular boundaries, separately. More importantly, in this analysis, our focus is the on and off of the boundaries.*

Figure 4c is confusing. + it's not clear how they calculated the expected counts ("permutation control") + if the x-axis is showing the scSB classes shouldn't the label be "# of cells"? + it's unclear what the y-axis refers to. Why "bins" and not "boundaries"?

*Answer: Thanks for the comment.*

*This is how the permutation control is calculated. By For each cell, we randomly sampled an identical number of boundaries from the cell, as one sample of the TAD-like domain boundaries. We repeated this process 1000 times and got the distribution of expected number of scSB-1, scSB-2, scSB-m and Absent in those 1000 samples.*

*We thank the reviewer pointing out the typo in Figure 4C for the x-axis labeling issue. The x-axis indeed represents the classes of scSBs; thus, it should be marked as # of cells. We corrected it in this revision.*

*In the deTOKI prediction, a boundary of TAD-like domain is exactly a bin; thus, bin and boundary are considered equivalent when boundary is intended. However, in Figure 4C, the column named Absent represents bins not predicted as boundaries in any single cells and thus should not be regarded as boundaries. For that reason, we annotated the y-axis as the # of bins.*

Figures 4d-e and 4f-g are hard to read and validate their claims. + The y-scales differ and the lines are barely distinguishable. They should have a panel for each histone mark with 2 lines for those that differ between conditions. + Perhaps they should consider using a package like Deeptools if it's applicable.

*Answer: Thanks for the suggestion.*

*We have redrawn Figure 4d, e and f following the reviewer’s suggestion (see PDF version). The Y-axis has been rescaled into a uniform range, i.e., 0.8-1.6 for all four panels so that the curves can be comparable. We moved most of the other histone markers into Supplementary Figure S7, leaving only H3K27me3 and H3K4me1 in the main Figure. With those modification, the trend now becomes clear. That is, the distribution of histone marks showed enrichment or depletion at the scSBs, similar to the ensemble TAD boundaries, e.g., H3K27me3 and H3K4me1 were enriched and depleted around the boundaries in single cells, respectively (Fig.4d and e; Supplementary Fig. 7a and b). However, this pattern tended to fluctuate with larger variation around the ensemble boundaries (Supplementary Fig.7d).*

It's unclear how they calculated the correlation of methylation pattern between inter and intra TAD bins. - how did they align the bins - how did the separate TAD boundaries into weak and strong (thresholds). - it would be interesting to see a figure of correlation or difference in methylation level as a function of TAD boundary.

*Answer: Thanks for the comment.*

*The correlation of methylation pattern between inter and intra TAD bin pairs was calculated as follows. The “inter TAD” indicates that the two bins are separated by a predicted TAD domain boundary, while the “intra TAD” indicates that the two bins belong to the same predicted TAD. To align bins, we pair the bins separated by 240Kb. A fixed distance was used to minimize the distance effect of chromatin contacts. This has been described in the figure legend.*

*The definition of weak and strong ensemble TAD boundaries was based on the order of insulation scores as calculated with Li’s data. The calculation was based on the pooled contact matrix of Li’s 150 single-cell Hi-C data. The TAD boundaries were sorted into descending order according to the calculated IS, and the first and last half were defined as weak and strong boundaries, respectively. To show a clearer trend of the correlation between the methylation level of inter TAD bin pairs and TAD boundaries, we added a new plot into Figure S8d, which further separated the boundaries into five groups according to the fractile quantiles. In this newly added plot, the correlation between the two were clearly shown. We copied this plot below.*

Algorithm Comparisons

The authors only compared their method with methods developed for ensemble HiC. They should consider including some methods specifically developed for single cell TADs like scHiCluster.

*Answer: Thanks for the comment.*

*We did not compare with scHiCluster in the previous version because the scHiCluster was not designed for TAD detection per se. The main advantage of scHiCluster was the imputation step it employed, and TADs were further called by a third-party tool named TopDom on the imputated data. We mentioned this type of tool in the discussion part of last version. Higashi is a tool submitted in bioRxiv rather recently. It also employed the imputation strategy and called TADs with another existing method.*

*As both reviewers have brought up this question, we compared the performance with scHiCluster on downsampled, simulated data and experimental data and also found that deTOKI outperformed scHiCluster (see revised Figure 2, 3). We also compared the performance with Higashi and found deTOKI is more efficient on TAD-like domain identification with single cell Hi-C data in terms of CPU running time. We added a new section in the supplementary text for this assessment, as also copied below.*

*To assess the improvement that data imputation may introduce to TAD-like domain predictions, we compared the performance of deTOKI, deTOKI with data imputation by scHiCluster, denoted as deTOKI+scHiCluster, and Higashi.*

*First, we compared the three tools with the downsampled 1/800 data of Dixon et al. and found comparable performance among the three. The largest absolute log2 fold changes (|log2FC|) in predicted TAD-like domain numbers between the downsampled datasets were all less than 0.3, and deTOKI+scHiCluster had the lowest |log2FC| on average (Supplementary Fig.10a) Taking the TADs identified with the full data as the gold standard, we compared the adjusted mutual information (AMI) and weighted similarity (WS) among the tools. All three tools had AMI and WS values rather close to each other with AMIs all around 0.8, on average, and WS about 0.71 (deTOKI+scHiCluster and Higashi were slightly better with average WS=0.74, Supplementary Fig.10b).*

*Second, we compared the three tools with simulated single-cell Hi-C data with setting identical to that in the main text between the genome region of chr18:50-55Mb and 10-15Mb (Supplementary Fig.10c). For all four indices assessed (AMI, WS, VI and BP), we found that the three tools performed in a similar manner. In the region chr18: 50-55Mb, deTOKI and deTOKI+scHiCluster showed better performance on all four indices than Higashi. In the region chr18: 10-15Mb, Higashi and deTOKI+scHiCluster had better performance than deTOKI alone in about half the settings. Last, for the classification task, deTOKI+scHiCluster had the lowest misclassification number compared to Higashi or deTOKI alone (Supplementary Fig.10d).*

*Taken together, deTOKI and Higashi have similar performance and deTOKI can be further improved by data imputation, e.g., scHiCluster. Considering the substantial CPU time required by Higashi (about 100-fold more CPU time than that required by either deTOKI or scHiCluster; Supplementary Table5), deTOKI is more efficient on TAD-like domain identification with single-cell Hi-C data.*

TADs have a hierarchical/nested structure. The author should discuss how their method deals with this. It seems that by exploring the structure of the coefficient matrix and/or the search for local maxima of strength they could tune the result to different resolutions.

*Answer: Thanks for the comment.*

*As the reviewer pointed out, deTOKI is runnable with different binsize. We tested the performance with 20kb and 80kb (Fig. S3) and found that deTOKI performed equally well with 40kb. However, we suspect that it may not be suitable for hierarchical structure in single cells with current single-cell Hi-C technologies.*

*The nature underlying domain hierarchy remains largely unexplored. Moreover, we cannot say for certain that the TAD-like domains we predicted are TADs. Therefore, it is difficult to claim that a hierarchical structure does or does not exist for those predicted TAD-like domains in single cells. Further, deTOKI was designed is to predict domain structure at the size of about 1Mb. This was mainly because of the ultra-sparse nature of single-cell Hi-C data, increasing unreliability for structures smaller than 100kb, as only a few reads can be mapped into those regions. Thus, we did not discuss the hierarchical structures in single cells.*

Have they experimented with using k-means or modularity clustering instead of NMF? k-means can be thought of as a form of NMF and it could speed up the process. It also eliminates the need of using a heuristic to assign bins to clusters.

*Answer: Thanks for the comment.*

*We tested the performance k-means or modularity clustering as alternatives to NMF. We found that K-means did not speed up the process, as both NMF and k-means spent about 0.15-0.2 seconds for a 200*200 single-cell Hi-C contact matrix (Python, sklearn, cluster number = 9). Further, k-means produced some suspicious domains in the tested data. As an example, for the domains in the left panel below, we have circled a domain predicted by k-means (in green) and divided it into two obvious data clusters. Last, we tested modularity clustering with the Louvain clustering algorithm in sknetwork lib in Python. We also found a suspicious domain (right panel below in green). Considering these results, the use of k-means or modularity clustering for domain prediction with single-cell HI-C data may require more specific tuning.*

Issues with the analysis.

In addition to the comments about the readability of the figures that make the claims hard to validate.

It is unclear what is the significance of the enrichment analysis of popular vs unpopular boundaries. If they want to claim that these boundaries have a functional role they should also include a GO analysis for the other (middle of the pack) TADs. If they want to claim that the distribution is non-random vs the scSB then they should show that these are not enriched in any term.

*Answer: Thanks for the comment.*

*The existence of popular and unpopular boundaries was tested by the Binomial test. We wanted to show that both popular and unpopular boundaries were not distributed randomly in a cell population, implying some functionality. GO analysis was just one possible approach that might help to reveal such functional implications, as the general connection between TADs and biological function remains elusive. Nevertheless, we performed the GO analysis on both popular, unpopular boundaries and those in the middle. We found a different set of GO terms enriched in all three groups (see Figure S6d-f).*

The definition of "unnested" TADs is not intuitive nor present in the reference. Moreover, they do not provide amble justification for the thresholds they used. They should show a plot of the strength of cross-boundary interactions vs the number of cells per TAD to demonstrate the overall relationship of the 2.

*Answer: Thanks for the comment.*

*The definition of nested and unnested TAD was borrowed from Beagan et al. (2020), whom we cited as reference 26. Briefly, nested and unnested TADs were those having many and a few cross-boundary contacts, respectively, as demonstrated in Figure 1a and 1c in Beagan et al. and copied below. In our work, we used a threshold for the top 20 and bottom 20 TADs as nested and unnested TADs, respectively. However, our conclusion is not sensitive to this threshold. For example, when we change the threshold to the top and bottom 40 TADs, we can draw similar conclusion (see Fig. S6b and also copied below). As the reviewer requested, we draw a plot of the strength of cross-boundary interactions vs. the number of cells per TAD, and a clear negative correlation can be seen (see Fig. S6c and below).*

The conclusion (lines 3-6 in p. 18) is not obvious from the figure 4c. The figure shows a steady drop in the number of bins/boundaries so why "few bins were taken as boundary in only one or two cells"?

*Answer: Thanks for the comment.*

*In agreement with the reviewer, we have revised as follows:*

*“Compared to permutated controls, far more scSBs in the scSB-m class were found and far fewer scSBs in the scSB-1 and scSB-2 classes (P<1E-4, Fig.4C). Moreover, bins not taken as boundaries for any cells in our data were also more prevalent than permutated controls (“Absent” in Fig. 4C). These results imply that the bins are either deficient or relatively prevalent, i.e., either with too few or too many cells to function as domain boundaries, respectively.”*

MNPPB is unnormalized with respect to the number of peaks in each dataset. Does this affect the comparison?

*Answer: Thanks for the comment.*

*Let’s discuss this issue regarding different figures separately.*

*Fig.3b and Fig.3c do not need normalization as all panels were generated with the same ChIP-seq data. This is also the case for Fig. S3d and Fig. S7f.*

*For Fig.4d-g, we normalized MNPPB by the average number of histone mark in the whole genome. We have mentioned this in the figure legend. This is also the case for Fig.S7a-c.*

Probable Typos

*paragraph from p.6 line 21 to p.7 line 13: they probably mean "i.e." instead of "e.g."

*page 30: the term N is used before it's defined.

*page 32: the denominator of T_{i,j} should probably be sum |S_ik * Sjk| otherwise T_{i,j} = 1.

*Answer: Thanks for the comment. We have correlated those typos.*

**Second round of review**

**Reviewer 2**

No further comments.
